# MS0621, a novel small-molecule modulator of Ewing sarcoma chromatin accessibility, interacts with an RNA-associated macromolecular complex and influences RNA splicing

**DOI:** 10.3389/fonc.2023.1099550

**Published:** 2023-01-30

**Authors:** Tamara Vital, Aminah Wali, Kyle V. Butler, Yan Xiong, Joseph P. Foster, Shelsa S. Marcel, Andrew W. McFadden, Valerie U. Nguyen, Benton M. Bailey, Kelsey N. Lamb, Lindsey I. James, Stephen V. Frye, Amber L. Mosely, Jian Jin, Samantha G. Pattenden, Ian J. Davis

**Affiliations:** ^1^ Curriculum in Genetics and Molecular Biology, University of North Carolina at Chapel Hill, Chapel Hill, NC, United States; ^2^ Department of Genetics, School of Medicine, University of North Carolina at Chapel Hill, Chapel Hill, NC, United States; ^3^ Mount Sinai Center for Therapeutics Discovery, Department of Pharmacological Sciences, Tisch Cancer Institute, Icahn School of Medicine at Mount Sinai, New York, NY, United States; ^4^ Mount Sinai Center for Therapeutics Discovery, Department of Oncological Sciences, Tisch Cancer Institute, Icahn School of Medicine at Mount Sinai, New York, NY, United States; ^5^ Mount Sinai Center for Therapeutics Discovery, Department of Neuroscience, Tisch Cancer Institute, Icahn School of Medicine at Mount Sinai, New York, NY, United States; ^6^ Curriculum in Bioinformatics and Computational Biology, University of North Carolina at Chapel Hill, Chapel Hill, NC, United States; ^7^ Lineberger Comprehensive Cancer Center, School of Medicine, University of North Carolina at Chapel Hill, Chapel Hill, NC, United States; ^8^ Center for Integrative Chemical Biology and Drug Discovery, Division of Chemical Biology and Medicinal Chemistry, UNC Eshelman School of Pharmacy, University of North Carolina at Chapel Hill, Chapel Hill, NC, United States; ^9^ Indiana University Simon Comprehensive Cancer Center, Indiana University School of Medicine, Indianapolis, IN, United States; ^10^ Department of Biochemistry and Molecular Biology, Indiana University School of Medicine, Indianapolis, IN, United States; ^11^ Department of Pediatrics, School of Medicine, University of North Carolina at Chapel Hill, Chapel Hill, NC, United States

**Keywords:** chromatin, RNA-processing, RNA-binding proteins, SWI/SNF (BAF) complex, Ewing sarcoma (ES), drug discovery

## Abstract

Ewing sarcoma is a cancer of children and young adults characterized by the critical translocation-associated fusion oncoprotein EWSR1::FLI1. EWSR1::FLI1 targets characteristic genetic loci where it mediates aberrant chromatin and the establishment of *de novo* enhancers. Ewing sarcoma thus provides a model to interrogate mechanisms underlying chromatin dysregulation in tumorigenesis. Previously, we developed a high-throughput chromatin-based screening platform based on the *de novo* enhancers and demonstrated its utility in identifying small molecules capable of altering chromatin accessibility. Here, we report the identification of MS0621, a molecule with previously uncharacterized mechanism of action, as a small molecule modulator of chromatin state at sites of aberrant chromatin accessibility at EWSR1::FLI1-bound loci. MS0621 suppresses cellular proliferation of Ewing sarcoma cell lines by cell cycle arrest. Proteomic studies demonstrate that MS0621 associates with EWSR1::FLI1, RNA binding and splicing proteins, as well as chromatin regulatory proteins. Surprisingly, interactions with chromatin and many RNA-binding proteins, including EWSR1::FLI1 and its known interactors, were RNA-independent. Our findings suggest that MS0621 affects EWSR1::FLI1-mediated chromatin activity by interacting with and altering the activity of RNA splicing machinery and chromatin modulating factors. Genetic modulation of these proteins similarly inhibits proliferation and alters chromatin in Ewing sarcoma cells. The use of an oncogene-associated chromatin signature as a target allows for a direct approach to screen for unrecognized modulators of epigenetic machinery and provides a framework for using chromatin-based assays for future therapeutic discovery efforts.

## Introduction

Cancer genotyping projects have repeatedly identified mutations in a broad range of epigenetic regulators (1, 2). The frequent identification of mutations in these genes highlights the importance of dysregulated chromatin in cancer. Unlike genetic alterations, chromatin state changes may be reversed, suggesting that they may be attractive therapeutic targets. Ewing sarcoma, an aggressive bone and soft tissue tumor of children and young adults, is characterized by a chromosomal rearrangement that generates a fusion oncoprotein central to the development and persistence of the cancer (3, 4). Other mutations, including STAG2 and TP53, are detected in a subset of tumors. In most cases, the translocation results in a chimeric oncoprotein, EWSR1::FLI1, that fuses the amino terminus of the RNA binding protein EWSR1 to the carboxy terminal DNA binding domain of the ETS family transcription factor FLI1 (3, 4).

Although the activities of EWSR1::FLI1 are not fully understood, several critical fusion-associated neomorphic features have been identified. Unlike the parental FLI1 transcription factor that contributes its DNA binding domain to the chimeric oncoprotein, EWSR1::FLI1 is largely retargeted to microsatellite repeats of the core ETS motif, (GGAA)n (5, 6). Targeting of EWSR1::FLI1 to these GGAA microsatellites results in recruitment of the SWI/SNF chromatin remodeling complex and p300 histone acetyltransferase, aberrant chromatin accessibility, and the establishment of *de novo* enhancers through which EWSR1::FLI1 activates the expression of many genes shown to be necessary for Ewing sarcoma proliferation (7, 8). The critical dependence on EWSR1::FLI1 and its exclusive expression in tumor cells makes it an attractive candidate for drug development. However, biochemical properties make small molecule pharmacological targeting of transcription factors challenging. We hypothesized that modulating an activity dependent on EWSR1::FLI1 would offer a strategy for relevant small molecule discovery. We developed a high-throughput, target-agnostic, chromatin-based screening platform to identify small molecules capable of altering chromatin states specifically at EWSR1::FLI1-bound GGAA microsatellites.

We previously reported the results of our screen which identified several modulators of EWSR1::FLI1-mediated chromatin states from a curated library consisting of chemical probes and tool compounds generated during structure activity relationship (SAR) studies targeted toward chromatin modulating proteins (9). In addition to several histone deacetylase inhibitors which we found acted by attenuating EWSR1::FLI1 expression, the screen identified MS0621, a previously uncharacterized molecule. Applying biochemical, genomic, and proteomic studies, we demonstrate that MS0621 arrests cell proliferation, interacts with EWSR1::FLI1 and numerous RNA binding proteins, and influences gene expression and RNA splicing.

## Materials and methods

### Cell culture

EWS894 and EWS502 cells were cultured in RPMI-1640 supplemented with 15% FBS. A673, A673-dervied cell lines, MHH-ES-1, and SU-CCS-1 cells were cultured in RPMI-1640 supplemented with 10% FBS. SK-N-MC cells were cultured in DMEM supplemented with 10% FBS, 2 mM L-glutamine, and 1X non-essential amino acids. TC-32 cells were cultured in RPMI-1640 supplemented with 10% FBS and 2mM L-glutamine. 786-O and UMRC2 cells were cultured in DMEM supplemented with 10% FBS. RPTEC cells were cultured using the REGM™ BulletKit™ (Lonza). HUVEC cells were cultured in the EGM™-2 BulletKit™ (Lonza) supplemented with 10% FBS. All cell lines were maintained at standard growth conditions of 37°C and 5% CO_2_. To assess cell proliferation, cells cultured in 96-well plates were assessed by WST-1 (Roche) according to manufacturer’s instructions or live cell imaging (Incucyte, S3). WST-1 Absorbance at 450 nM was measured (Cytation 5, Agilent BioTek). To assess growth in soft agar, cells were suspended in 0.5% low melting point agarose, 1X RPMI, 15% fetal bovine serum at a density of 4500 cells per well and layered over one mL of base agar (0.6% agarose, 1X RPMI, 15% fetal bovine serum) in a 6-well dish. MS0621 or DMSO was diluted in top agar layer to desired final concentration. Plates were overlayed with additional RPMI containing compound on day 5 and day 11. Plates were stained with MTT (0.5 mg/ml) on day 15 to visualize cell colonies.

### Chemistry general procedures

All chemical reagents were purchased from commercial vendors and used in syntheses without further purification. An Agilent 1200 series system with a DAD detector and a 2.1 mm × 150 mm Zorbax 300SB-C18 5 μm column with water containing 0.1% formic acid as solvent A and acetonitrile containing 0.1% formic acid as solvent B at a flow rate of 0.4 mL/min for chromatography were used to obtain high performance liquid chromatography (HPLC) spectra for all final compounds. The gradient program was as follows: 1% B (0−1 min), 1−99% B (1−4 min), and 99% B (4−8 min). Chromatography was performed using a 2.1 mm × 30 mm ACQUITY UPLC BEH C18 1.7 μm column with water containing 3% acetonitrile and 0.1% formic acid as solvent A and acetonitrile containing 0.1% formic acid as solvent B at a flow rate of 0.8 mL/min. High-resolution mass spectra (HRMS) data were obtained in positive ion mode using an Agilent G1969A API-TOF with an electrospray ionization (ESI) source. Nuclear Magnetic Resonance (NMR) spectra were obtained on a Bruker DRX-600 spectrometer with 600 MHz for proton (^1^H NMR) 150 MHz for carbon (^13^C NMR) or a Varian Mercury spectrometer at 400 MHz for proton (^1^H NMR), 100 MHz for carbon (^13^C NMR); chemical shifts are reported in ppm (δ). Preparative HPLC was performed using Agilent Prep 1200 series with a UV detector set to 254 nm. Samples were injected into a Phenomenex Luna 75 mm × 30 mm, 5 μm, C18 column at room temperature. The flow rate was 40 mL/min. A linear gradient was used with 10% of MeOH (A) in H2O (with 0.1% TFA) (B) to 100% of MeOH (A). HPLC and UPLC were used to establish the purity of target compounds. All final compounds had >95% purity using the HPLC and UPLC methods described above.

**Scheme 1:**
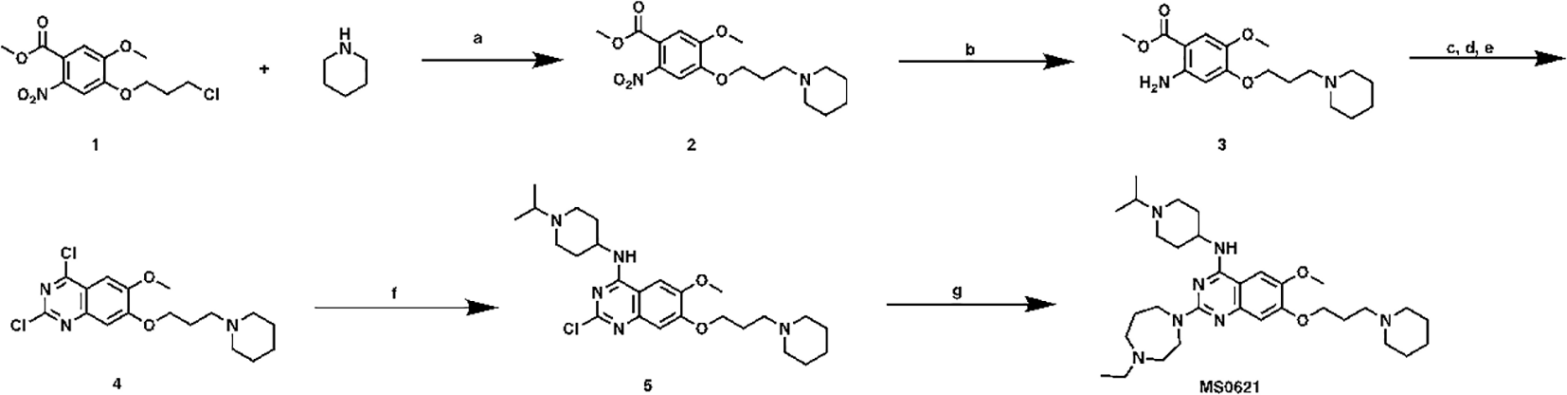
Synthetic route for MS0621 Reagents and conditions: **(A)** Piperidine, K_2_CO_3_, NaI, CH_3_CN, 80°C, overnight, 78%; **(B)** Fe, NH_4_OAc, EtOAC/H_2_O, reflux, overnight, 58%; **(c)** NaOCN, HOAc/H_2_O, rt, overnight; **(D)** NaOH, MeOH/H_2_O, reflux, 3h; **(E)** POCl_3_, PhEt_2_, reflux, 7h, 50%; **(F)** (1-isopropylpiperidin-4-yl)amine, DIEA, THF, rt, overnight, 99%; **(G)** 1-Ethylhomopiperazine, TFA, *i*-PrOH, MW, 160°C, 15 min, 80%.

2-(4-ethyl-1,4-diazepan-1-yl)-*N*-(1-isopropylpiperidin-4-yl)-6-methoxy-7-(3-(piperidin-1-yl)propoxy)quinazolin-4-amine (MS0621, **Scheme1**) To a solution of compound 1 (6.9 g, 22.6 mmol) in CH_3_CN (60 mL) were added piperidine (6.9 mL, 70 mmol), K_2_CO_3_ (6.9g, 50 mmol) and NaI (6.8g, 45 mmol). After heated at 80°C overnight, the mixture was cooled down to room temperature followed by filtered. The filtrate was collected, concentrated and purified by ISCO to yield compound 2 as brown oil (6.2 g, 78% yield). ^1^H NMR (400 MHz, Chloroform-*d*) δ 7.41 (s, 1H), 7.00 (s, 1H), 4.09 (t, *J* = 6.6 Hz, 2H), 3.89 (s, 3H), 3.84 (s, 3H), 2.41 (t, *J* = 7.2 Hz, 2H), 2.37 – 2.26 (m, 4H), 2.04 – 1.91 (m, 2H), 1.56 – 1.48 (m, 4H), 1.44 – 1.31 (m, 2H).

Compound 2 (6.2 g, 17.6 mmol), Fe powder (3.9 g, 70 mmol) and NH_4_OAc (8.2 g, 106 mmol) were mixed in EtOAC/H_2_O (1:1, 100 mL). The mixture was heated reflux overnight followed by filtered. The filtrate was washed with brine (50 mL), dried and concentrated to yield compound 3 as brown oil (3.3 g, 58% yield). ^1^H NMR (400 MHz, Chloroform-*d*) δ 7.19 (s, 2H), 3.99 (t, *J* = 6.6 Hz, 2H), 3.78 (s, 3H), 3.73 (s, 3H), 2.58 – 2.51 (m, 2H), 2.51 – 2.40 (m, 4H), 2.10 – 1.97 (m, 2H), 1.65 – 1.53 (m, 4H), 1.46 – 1.35 (m, 2H).

Compound 3 (3.3 g, 10.2 mmol) and NaOCN (1.0 g,15.4 mmol) were dissolved in HOAc/H_2_O (2:1, 30 mL), and stirred at room temperature overnight. Then the solvent was removed, the resulting residue was re-dissolved in MeOH (30 mL) followed by NaOH solution (10%, 10 mL). After heated at reflux for 3 h, the mixture was cooled down, and neutralized with conc. HCl to give the precipitate. The solid was collected and dried. The crude product was dissolved in POCl_3_ (30 mL) together with PhNEt_2_ (1.0 mL, 6.1 mmol). The mixture was heated at reflux for 7 h, the excess solvent was removed. The resulting residue was treated with ice followed by sat. NaHCO_3_ solution until no gas was released. The mixture was extracted with DCM (3 × 30 mL), and organic layer was collected, dried and purified by ISCO to yield compound 4 as yellow solid (1.9 g, 50% yield in 3 steps). ^1^H NMR (400 MHz, Chloroform-*d*) δ 7.33 (s, 1H), 7.28 (s, 1H), 4.24 (t, *J* = 6.6 Hz, 2H), 4.03 (s, 3H), 2.50 (t, *J* = 7.2 Hz, 2H), 2.42 – 2.38 (m, 4H), 2.17 – 2.05 (m, 2H), 1.64 – 1.54 (m, 4H), 1.49 – 1.38 (m, 2H).

To a solution of Compound 4 (140 mg, 0.38 mmol) in THF (3 mL), were added DIEA (0.13 mL, 0.76 mmol) and (1-isopropylpiperidin-4-yl)amine (160 mg, 1.13 mmol). After stirring at room temperature overnight, the solution was concentrated and purified by ISCO to yield compound 5 as brown solid (140 mg, 95% yield). ^1^H NMR (400 MHz, Chloroform-*d*) δ 7.06 (s, 1H), 6.79 (s, 1H), 4.32 (s, 1H), 4.10 (t, *J* = 6.6 Hz, 2H), 3.93 (s, 3H), 3.11 – 2.96 (m, 3H), 2.55 (t, *J* = 11.7 Hz, 2H), 2.47 (t, *J* = 7.4 Hz, 2H), 2.40 – 2.36 (m, 4H), 2.17 (d, *J* = 12.9 Hz, 2H), 2.09 – 1.98 (m, 2H), 1.94 – 1.86 (m, 2H), 1.58 – 1.49 (m, 4H), 1.42 – 1.33 (m, 2H), 1.15 (d, *J* = 6.6 Hz, 6H).

A mixture of the compound 5 (75 mg, 0.16 mmol) 1-ethylhomopiperazine (41 mg, 0.32 mmol), and TFA (72 mg, 0.64 mmol) in *i*-PrOH (0.26 mL) was heated by microwave irradiation to 160 °C for 15 min in a sealed tube. After concentration *in vacuo*, the crude product was purified by preparative HPLC to yield MS0621 in TFA salt form. The resulted product was basified with saturated aq. NaHCO_3_ and extracted with CH_2_Cl_2_ to afford the titled compound as a white solid (71 mg, 80% yield). ^1^H NMR (400 MHz, CDCl_3_) δ 6.87 (s, 1H), 6.72 (s, 1H), 4.98 (d, *J* = 7.1 Hz, 1H), 4.12 (t, *J* = 6.8 Hz, 2H), 4.08 – 3.98 (m, 1H), 3.98 – 3.91 (m, 2H), 3.90 – 3.80 (m, 5H), 2.92 – 2.84 (m, 2H), 2.79 – 2.68 (m, 3H), 2.65 – 2.57 (m, 2H), 2.54 (q, *J* = 7.1 Hz, 2H), 2.43 (t, *J* = 7.2 Hz, 2H), 2.40 – 2.23 (m, 6H), 2.21 – 2.10 (m, 2H), 2.08 – 1.91 (m, 4H), 1.61 – 1.48 (m, 6H), 1.45 – 1.36 (m, 2H), 1.13 – 0.98 (m, 9H). ^13^C NMR (100 MHz, CDCl_3_, two overlapping peaks) δ 158.73, 158.12, 154.05, 149.80, 145.20, 107.06, 102.78, 101.70, 67.51, 56.76, 55.93, 55.86, 54.66(2C), 54.58, 54.45, 51.67, 48.77, 47.89(2C), 46.28, 45.86, 32.76(2C), 27.98, 26.60, 26.11(2C), 24.57, 18.60(2C), 12.51. HRMS calculated for C_32_H_53_N_7_O_2_ [M+H]^+^: 568.4339. Found: 568.4350.

**Scheme 2:**
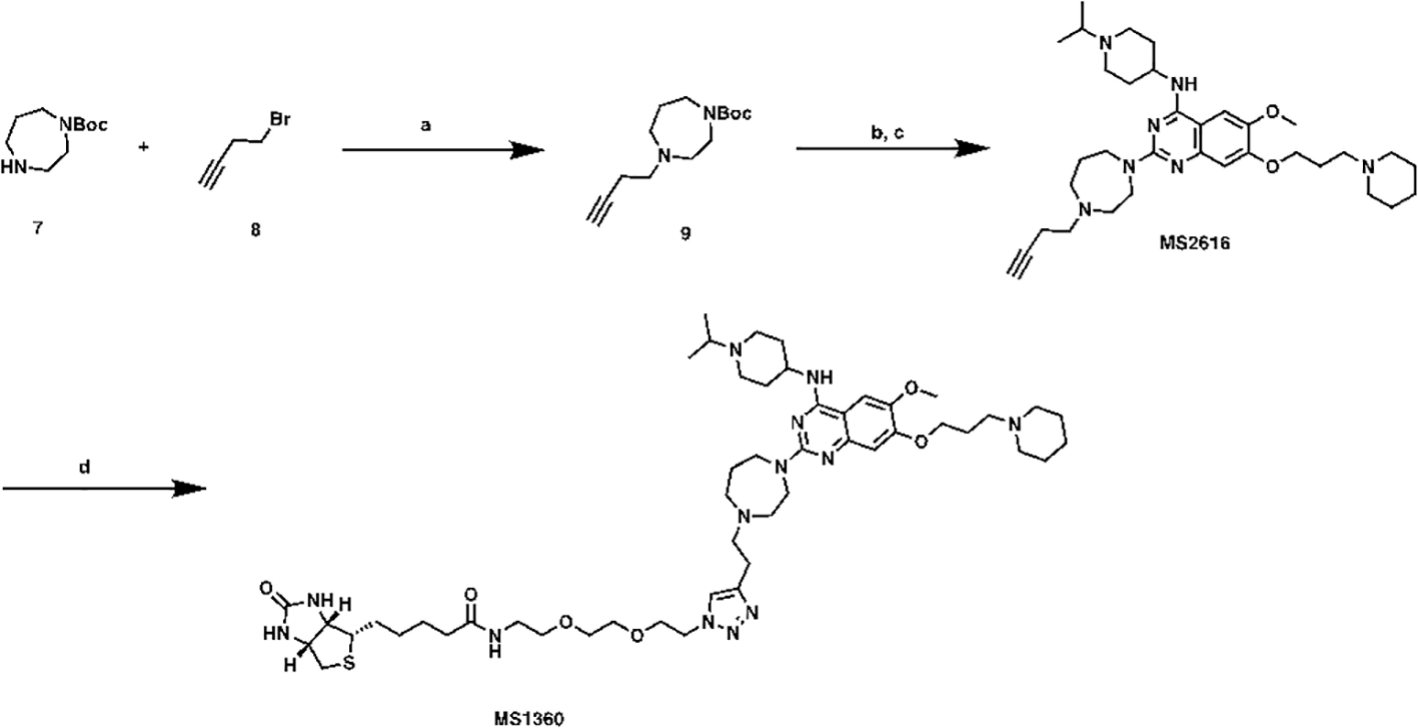
Synthetic route for MS1360 Reagents and conditions: **(A)** K_2_CO_3_, CH_3_CN, reflux, 5 h, 56%; **(B)** TFA, DCM, rt; **(C)** TFA, *i*-PrOH, MW, 130°C, 20 min, 40% in 2 steps. **(D)** azide-PEG2-biotin conjugate, Vc, CuSO_4_, *t*-BuOH, rt, overnight, 40%.

*N*-(2-(2-(2-(4-(2-(4-(4-((1-isopropylpiperidin-4-yl)amino)-6-methoxy-7-(3-(piperidin-1-yl)propoxy)quinazolin-2-yl)-1,4-diazepan-1-yl)ethyl)-1*H*-1,2,3-triazol-1-yl)ethoxy)ethoxy)ethyl)-5-((3a*S*,4*S*,6a*R*)-2-oxohexahydro-1*H*-thieno[3,4-*d*]imidazol-4-yl)pentanamide (MS1360, **Scheme2**). To a commercial available compound 7 (210 mg, 1.1 mmol) in CH_3_CN (5 mL), was added compound 8 (0.11 mL, 1.2 mmol) followed by K_2_CO_3_ (200 mg, 1.4 mmol). After stirring at reflux for 5 h, the mixture was cooled down and filtered, filtrate was collected and concentrated. The resulting residue was purified by ISCO to yield compound 9 as brown oil (150 mg, 56% yield). ^1^H NMR (600 MHz, Chloroform-*d*) δ 3.56 – 3.39 (m, 4H), 2.77 (d, *J* = 7.8 Hz, 2H), 2.75 – 2.65 (m, 5H), 2.43 – 2.31 (m, 2H), 1.89 – 1.77 (m, 2H), 1.48 (s, 9H).

Compound 9 (150 mg, 0.6 mmol) was dissolved in DCM/TFA (2:1, 3 mL). after stirred at room temperature for 1 h, the excess solvent was removed, the resulting residue was re-dissolved in *i*-PrOH (1 mL), followed by compound 5 (148 mg, 0.31mmol) and TFA (0.1 mL). The result solution was heated by microwave irradiation to 130 °C for 20 min in a sealed tube. After concentration *in vacuo*, the crude product was purified by preparative HPLC to yield MS2616 as in TFA salt form. MS2616 was basified with saturated aq. NaHCO_3_ and extracted with CH_2_Cl_2_ to afford free base form of MS2616 as a brown solid (73 mg, 40% yield). ^1^H NMR (600 MHz, Methanol-*d*_4_) δ 7.43 (s, 1H), 6.91 (s, 1H), 4.13 – 4.04 (m, 3H), 3.94 – 3.87 (m, 5H), 3.84 (t, *J* = 6.3 Hz, 2H), 3.01 – 2.92 (m, 2H), 2.85 (t, *J* = 5.0 Hz, 2H), 2.77 – 2.70 (m, 3H), 2.67 (t, *J* = 5.5 Hz, 2H), 2.55 – 2.49 (m, 2H), 2.49 – 2.33 (m, 6H), 2.33 – 2.25 (m, 2H), 2.16 (s, 1H), 2.15 – 2.08 (m, 2H), 2.06 – 1.92 (m, 4H), 1.74 – 1.64 (m, 2H), 1.64 – 1.56 (m, 4H), 1.51 – 1.39 (m, 2H), 1.10 (d, *J* = 6.6 Hz, 6H). HRMS calculated for C_34_H_54_N_7_O_2_ [M+H]^+^: 592.4334. Found: 592.4324.

MS2616 (25 mg, 0.04 mmol), azide-PEG3-biotin conjugate (16 mg, 0.04 mmol), Vc (0.1M in water, 0.1 mL, 0.01 mmol) and CuSO_4_ (0.1 M in water, 0.1 mL, 0.01 mmol) were mixed in *t*-BuOH (1 mL). After stirring overnight, the mixture was purified by prep-HPLC to yield titled compound as brown oil (16 mg, 40% yield). ^1^H NMR (600 MHz, Methanol-*d*_4_) δ 7.99 (s, 1H), 7.79 (s, 1H), 7.30 (s, 1H), 4.63 – 4.56 (m, 3H), 4.51 (dd, *J* = 7.9, 4.8 Hz, 1H), 4.44 – 4.14 (m, 5H), 4.10 – 3.93 (m, 5H), 3.93 – 3.84 (m, 3H), 3.79 – 3.53 (m, 15H), 3.50 (t, *J* = 5.6 Hz, 2H), 3.38 (d, *J* = 5.8 Hz, 2H), 3.36 – 3.31 (m, 4H), 3.31 – 3.25 (m, 2H), 3.24 – 3.18 (m, 1H), 3.00 (td, *J* = 12.6, 3.0 Hz, 2H), 2.94 (dd, *J* = 12.7, 5.0 Hz, 1H), 2.71 (d, *J* = 12.7 Hz, 1H), 2.55 – 2.40 (m, 4H), 2.38 (dq, *J* = 11.7, 6.0 Hz, 2H), 2.21 (t, *J* = 7.4 Hz, 2H), 2.15 – 2.05 (m, 2H), 2.05 – 1.96 (m, 2H), 1.93 – 1.87 (m, 1H), 1.87 – 1.77 (m, 2H), 1.77 – 1.68 (m, 1H), 1.67 – 1.52 (m, 4H), 1.48 – 1.36 (m, 8H). HRMS calculated for C_50_H_82_N_13_O_6_S [M+H]^+^: 992.6226. Found: 992.6256.

### Preparation and lentiviral infection of cell lines

Lentivirus was produced by transfection of HEK293-T cells with constructs and packaging vectors (pVSVG, pRRE, pRSV) as described (10). Supernatant was collected over 48 h and concentrated with Lenti-X Concentrator (Clontech). Cells were infected with concentrated lentivirus in the presence of polybrene (10 μg/mL). Media was replaced 24 hours after initial infection.

### Generation of CRISPRi cell lines

The A673-CRISPRi and 502-CRISPRi cell lines stably expressing KRAB-dCas9 were generated using lentiviral infection and drug selection (11). Lentivirus for KRAB-dCas9 was produced as described above. 48 hours after infection with KRAB-dCas9 lentivirus, cells were selected with Blasticidin for three weeks. Single guide RNA (sgRNA) expression vectors were generated by ligating oligonucleotides into the VDB783 vector which had been digested with AarI (**Table 1**). Lentivirus for each sgRNA was produced as described above.

**Table 1:** sgRNAs used in these studies.

sgRNA Sequences	
Target	Sequence 5’ to 3’
EWSR1	GCGCGAGGACCGCCACACAA
hnRNPH1	GGCAAAAACGGTACCCACCG

### FAIRE-qPCR

FAIRE was performed as previously described (12). Briefly, cells were treated with MS0621 or vehicle control for 16 hours prior to fixation in 1% formaldehyde, quenching by the addition of glycine to a final concentration of 125 mM, washing in 1X PBS, and resuspension in 2 mL FAIRE Lysis Buffer (10 mM Tris HCl pH 8, 2% Triton X-100, 1% SDS, 100 mM NaCl, 1 mM EDTA). Cells were sonicated with a microtip (Misonix Sonicator 3000) to an average fragment size of 200 - 500 bp. Regions of nucleosome-depleted chromatin were isolated by organic extraction by phenol-chloroform or column (ChIP DNA Clean & Concentrator, Zymo Research). Chromatin was then subjected to RNaseA digestion (Sigma, 30 min at 37°C), proteinase K digestion (NEB, 1 hour at 55°C, then overnight at 65°C to reverse crosslinks). Input chromatin was subjected to the same RNase and proteinase K digestion prior to phenol-chloroform extraction. Finally, FAIRE and input chromatin were purified using the ChIP DNA Clean & Concentrator kit (Zymo). Each sample was quantified by RT-qPCR in triplicate (ViiA 7 Real-Time PCR system, Applied Biosystems) using iTaq Universal SYBR Green Supermix (Bio-Rad) in a total volume of 10 μL. Primer sequences are listed in **Table 2**. Percent input was determined using the ΔCt method (13). Relative chromatin inhibition was calculated as previously described (9).

**Table 2:** PCR primers used in these studies.

PCR Primers		
FAIRE and ChIP Primers		
Locus	Forward Sequence (5’-3’)	Reverse Sequence (5’-3’)
P1 (screening site)	AAGGAAGGAAGGGAGGGACACATAC	CCTGTGAGTGTGACAGATTACTTGG
P7 (screening site)	GGGTGACAGAGTAAGATCCTGTCAGA	TGGGCGTGGTTCTCATGT
AURKAIP1 (+ control)	TATACCCGCAGGTCCAGAATCGTT	AATAGCTCTAGACGCTTCCGCCTT
BC006361 (- control)	TTCTCCAACTTTGGAAGCCCAGGA	TGTCTCCTTCTAGGCCCTCACAAT
JAK1	GGGAGGAATTGAAAGGAAGTGTGT	TGTGAAACCTCGTCTGATCCACCCT
NR0B1	GCATCAGGAAGCCTGGATCCATTA	GTATATACCAACACCCTTCCCTG
CAV1	AAGGAAGGAAGGAAGACCCT	GTACAACGAATCCCTGTGACACAAA
MDM2	TGGATCTGAGGAGGAAATGTGCGT	TAACTCATCCCTGTGCCTCTGCT
JAK1.2	GGAGACAGGCTCCAACACAGGAAA	AAGTATCCCTCAAGTTGCCCTGCT
NKX2-2	TCTCTCCTTTCCATCGTTGGTGGT	AGCACTCATCCTTAAGCCTCAACC
Negative Site 1	TGGATGCACAGAGAATGTCCACCT	TGGTTCGTAAGTGACAGAGCCAGA
Expression Primers		
Target	Forward Sequence (5’-3’)	Reverse Sequence (5’-3’)
EWS-FLI	GCTATGGTCAACAAAGCAGCTATG	TTGGCTAGGCGACTGCTGGT
GAPDH	CTGACTTCAACAGCGACACC	TAGCCAAATTCGTTGTCATACC

For FAIRE in A673-CRISPRi cells, cells were infected with sgRNA lentivirus as described above. Five days post lentiviral infection, mCherry expression was assessed, and cells were harvested for FAIRE as described above.

### RNA extraction and qRT-PCR

Following 16-hour treatment with MS0621 or vehicle (PBS) cells were harvested in Trizol (Invitrogen). RNA was extracted and cDNA was prepared using SuperScript III First-Strand Synthesis SuperMix for qRT-PCR (Invitrogen) according to manufacturer’s protocols. Each sample was quantified by RT-qPCR in triplicate (ViiA 7 Real-Time PCR system, Applied Biosystems) using iTaq Universal SYBR Green Supermix (Bio-Rad) in a total volume of 10 μL. Primer sequences are listed in **Table 2**. Fold change was determined using the ΔCt method (13).

### ChIP-qPCR

Following 16-hour treatment with MS0621 or vehicle (PBS), cells were fixed in 1% formaldehyde and quenched with 125 mM glycine. Nuclei were isolated by hypotonic lysis (10mM Tris pH 7.4 15mM NaCl, 60mM KCl, 1mM EDTA, 0.1% NP-40, 5% sucrose, 1x protease inhibitors) and dounce homogenization. Nuclei were purified over a sucrose cushion (10 mM Tris HCl pH 7.4, 15 mM NaCl, 60 mM KCl, 10% Sucrose) and chromatin was sheared by sonication (Misonix Sonicator 3000) to an average fragment size of 200 bp – 1 kb. Chromatin was immunoprecipitated using 4 μg of FLI1 (Abcam ab133485) or IgG (Cell Signaling 2729S) antibody. Immunoprecipitated and input chromatin were incubated with RNaseA (Sigma, R4642) for 30 min at 37°C and proteinase K (NEB, P8107S) for 1 hour at 55°C followed by overnight crosslink reversal at 65°C before column purification (ChIP DNA Clean & Concentrate, Zymo, D5201). Quantitative PCR was performed (iTaq Universal SYBR Green Supermix, Bio-Rad, 1725124), using primers listed in **Table 2**.

### Apoptosis analysis

MS0621-treated or vehicle-treated EWS502 cells were prepared and stained using the FITC Annexin V Apoptosis Detection Kit according to manufacturer’s instructions (BD Biosciences, cat. No. 556547). Flow cytometry was performed immediately after staining (LSR II, BD Biosciences).

### Cell cycle analysis

MS0621 or vehicle treated EWS502 cells were washed with PBS and fixed with 70% ice cold ethanol, then stained with propidium iodide. Flow cytometry was performed immediately after staining, collecting 10,000 total events at 100-200 events per second (CyAN ADP, Beckman Coulter). For analysis of cell division, EWS502 cells were stained with CFSE (CellTrace CFSE Cell Proliferation Kit for flow cytometry, Thermo Fisher) followed by treatment with either MS0621 or vehicle control.

### BrdU incorporation analysis

MS0621-treated or vehicle-treated EWS502 cells were stained and fixed using the FITC BrdU Flow Kit (BD Biosciences, #559619) according to manufacturer’s instructions. BrdU pulse was performed on live cells 1 hour just before harvesting. Stained cells were analyzed by flow cytometry, collecting 10,000 total events at 100-200 events per second (CyAN ADP, Beckman Coulter).

### Micrococcal nuclease-digested nucleosome extract preparation

Nucleosome extracts were prepared as previously described (14). Briefly, cells were harvested by scraping, washed in PBS, and resuspended in Buffer A (10 mM HEPES pH 7.9, 10 mM KCl, 1.5 mM MgCl_2_, 340 mM sucrose, 10% glycerol supplemented with 0.5 mM PMSF, 5 mM 2-mercaptoethanol, 1X Roche protease inhibitor cocktail, and 1 nM SAHA). Cells were lysed by the addition of an equivalent volume of Buffer A supplemented with 0.2% Triton X-100. Nuclei were pelleted by centrifugation and resuspended in supplemented Buffer A. Cellular debris was removed by centrifuging the nuclei through a sucrose cushion (10 mM HEPES pH 7.9, 30% sucrose, 1.5 mM MgCl_2_, supplemented with 0.5 mM PMSF, 5 mM 2-mercaptoethanol, and 1X Roche protease inhibitor cocktail). Nuclei were treated with micrococcal nuclease (Worthington) in Buffer A with 1 mM CaCl_2_, following pre-incubation at 37°C for 5 minutes. Once only mononucleosomes remained, digestion was halted with EDTA to 1.3 mM on ice. Following centrifugation at 18,000 g for 5 min at 4°C protein concentration in the supernatant was measured (Rapid Gold BCA assay, Pierce/ThermoScientific) and extracts were diluted to 1 mg/mL for chemoprecipitation experiments.

### Chemiprecipitation and proteomics

Nuclear extracts were diluted to 1 mg/mL in Protein Binding Buffer (PBB, 150 mM NaCl, 50 mM Tris HCl pH 8.0, 0.1% NP-40) and incubated with UNC4151 biotinylated-analog for 2 hours at 4°C followed by capture on pre-washed MyOne T1 streptavidin beads (Invitrogen) for 2 hours at 4°C. Supernatant was removed, and beads were washed three times at 4°C in PBB followed by elution of bound proteins. For RNase digestion experiments, extracts pre-incubated with biotinylated compound were divided into two tubes, 100 μg/mL RNase A (Sigma) was added to one tube, and proteins from each tube were captured on T1 streptavidin beads. For western blotting, proteins were eluted in 1X loading buffer with 2-mercaptoethanol at 95°C for 5 min. For proteomics analyses, protein-bound beads were digested with trypsin. Digestions were quenched with 0.1% formic acid and peptides were pressure loaded onto a microcapillary column paced with strong cation and reverse phase resin. A 10-step MudPIT run was performed on an LTQ Velos Pro with in-line Proxeon Easy nLC. The 10 most intense ions identified in MS1 by the mass spectrometer in data dependent acquisition mode were selected for MS/MS fragmentation using collision induced dissociation. Dynamic exclusion was set to 90 sections with a repeat count of 1. Raw data files were matched to the protein database using SEQUEST (Proteome Discoverer 1.4, Thermo).

### Immunoprecipitation

Extracts (800 μg) were adjusted to a final volume of 350 μL in CSK Buffer (10 mM Pipes pH 7.0, 300 mM Sucrose, 100 mM NaCl, 3 mM MgCl_2_). Extracts were incubated with 2 μg of FLI1 (Abcam ab133485) or IgG (Cell Signaling 2729S) antibody at 4°C overnight followed by capture on prewashed SureBead Protein A magnetic beads for 1 hour at 4°C. Beads were washed three times in CSK Buffer at 4°C and once in 50 mM Tris-HCl pH 8.0. Proteins were eluted as previously described (15) with the following adjustments: beads were resuspended in IP Soft Elution Buffer (10 mM Tris-HCl pH 8.0, 0.2% SDS, 0.1 Tween-20) and incubated seven minutes at room temperature with regular vortexing. Supernatant was collected in a new tube, elution was repeated once, and eluates were pooled.

### RNA sequencing and analysis

Following 16-hour treatment with 5 μM MS0621 or vehicle (DMSO) EWS894 cells were washed in PBS and harvested in Trizol (Invitrogen). Poly-A selected sequencing libraries were generated using the KAPA Stranded mRNA-Seq Kit for Illumina platforms (KAPA Biosystems) according to manufacturer’s protocol and 50 bp paired-end sequencing was performed (Illumina, HiSeq 2500). Adaptor sequences were removed from reads using cutadapt (v.1.12). High quality reads were isolated using FASTX-Toolkit (v0.0.12) passing options -Q 33, -p 90, and q 20. Reads were aligned to the hg38 genome using STAR(v2.5.2b) with the following options: –quantMode TranscriptomeSAM, –alignIntronMax 1000000, –outFilterMismatchNmax 2, –alignIntronMin 20, –chimSegmentMin 15, –outSAMtype BAM Unsorted, –chimJunctionOverhangMin 15, –outFilterType BySJout, –outFilterScoreMin 1. GENCODE V41 GTF file was used for annotation. Gene expression estimates (TPM) were calculated using Salmon (v.1.6.0). Differentially expressed genes were identified using DESeq2 (16). Gene set enrichment analysis (GSEA) and gene ontology analysis were performed using the R package clusterProfiler (v.4.2.4) using the gseGO and enrichGO functions, respectively (17). The Molecular Signatures Database (MSigDB, v.7.5.1) C2-CBP gene set was used for GSEA and the “BP” subontology was used for gene ontology analysis.

Alternatively spliced genes were identified using rmats-turbo (v.4.1.1), with the option –readlength 50, the GENCODE V41 comprehensive gene annotation GTF file, and a STAR index for hg38-ERCC (18). Alternatively spliced transcripts were also identified using rmats-turbo with above settings using the GENCODE V41 (knownGene,limited) GTF file downloaded from UCSC Genome Browser. We further defined differentially spliced events as those that met all three of the following criteria: event supported by a minimum of 20 reads, absolute value of the Inclusion Level Difference greater than or equal to 10, and FDR less than 0.05. Shared skipped exon events were identified using the R package plyranges (v.1.14.0) using the join_overlap_inner_within function on exon coordinates (19). To assess enrichment of shared events, we permuted over all spliceable exons with minimum read coverage greater than or equal to 20 reads 1,000 times and computed a two-sided p-value. We define spliceable exons as any exon that is not the first, last, or only exon in a transcript. Read coverage over exons was computed using the deeptools (v.3.5.1) multiBamSummary function using the BED-file mode. Sashimi plots were generated using rmats2sashimiplot (v.2.0.4) using the argument –intron_s 3.

### Western blot

To assess EWSR1::FLI1 protein levels, cells were harvested in 2X SDS-loading buffer with 2-mercaptoethanol and boiled. To assess proteins enriched by immunoprecipitation, eluted proteins were diluted 1:1 in 2X SDS-loading buffer with 2-mercaptoethanol and incubated at 95°C for 5 min. For all western blots, proteins were resolved by SDS/PAGE (BioRad) and were transferred onto a nitrocellulose membrane (BioRad). After blocking in 5% BSA in PBS, membranes were probed using the appropriate primary antibody (**Table 3**) and fluorescent secondary antibodies (LiCor). Quantification was performed using Image Studio Lite (LiCor, version 5.2).

**Table 3:** Antibodies used in these studies.

Antibodies				
Target	Host	Manufacturer	Catalog#	Application
FLI	Rabbit polyclonal	Abcam	ab133485	WB/IP/ChIP
α-Tubulin	Mouse monoclonal	Sigma/EMD Millipore	T9026	WB
HA	Rabbit polyclonal	Abcam	ab9110	WB/IP
Ku80	Rabbit polyclonal	Genetex	GTX70485	WB
ATF1	Rabbit polyclonal	Bethyl	A303-034A	WB
Histone H3	Rabbit polyclonal	Sigma/EMD Millipore	07-690	WB
hnRNPC	Rabbit polyclonal	Proteintech	11760-1-AP	WB
YBX1	Rabbit polyclonal	Proteintech	20339-1-AP	WB
EWSR1	Rabbit polyclonal	Bethyl	A300-416A	WB
hnRNPH1	Mouse monoclonal	Proteintech	67375-1-Ig	WB
DHX9	Mouse monoclonal	Proteintech	67153-1-Ig	WB
ARID1A (BAF250A)	Rabbit monoclonal	Cell Signaling Technology	12354	WB
SMARCA4 (BRG1)	Rabbit monoclonal	Abcam	ab110641	WB
ARID1B	Mouse monoclonal	Abcam	ab57461	WB
Normal IgG	Rabbit	Cell Signaling	2729S	ChIP

## Results

### Identification of a chemical modulator of EWSR1::FLI1-mediated chromatin

**Figure 1:**
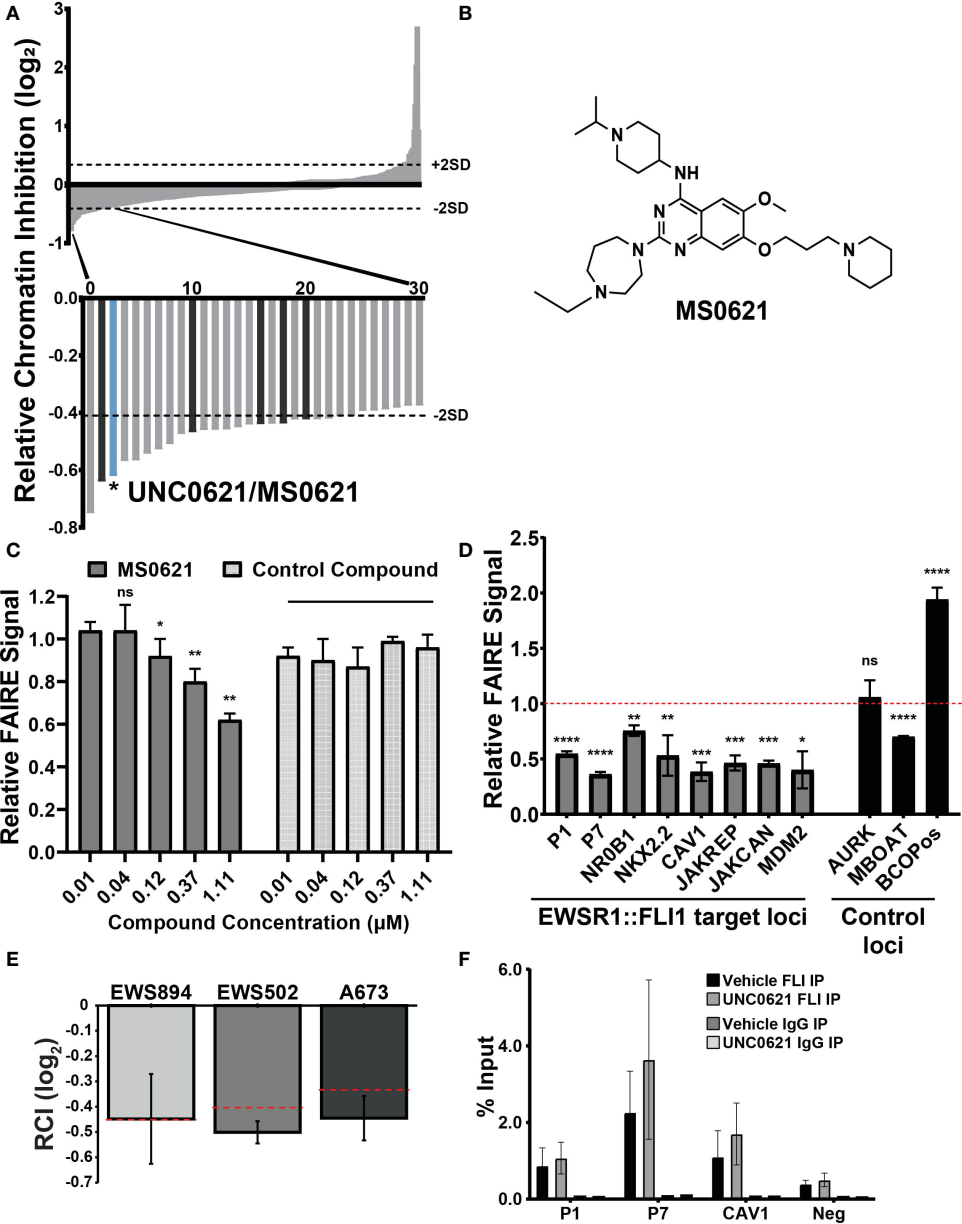
A chromatin-based screen identifies a small molecule modulator of chromatin state at EWS-FLI-bound GGAA microsatellites. **(A)** Waterfall plot of screen results with rank ordered Relative Chromatin Inhibition values (RCI, FAIRE signal at Ewing sarcoma regions/FAIRE at control regions, log2). Dashed lines indicate +/- 2SDs from the average RCI for vehicle controls. Magnification: Thirty compounds demonstrating the greatest decrease in FAIRE signal. The bar representing UNC0621/MS0621 is indicated in light blue. Dark gray bars indicate structural analogs of MS0621 (discussed further in **Figure 2**). Figure adapted from (9). **(B)** Chemical structure of MS0621 **(C)** FAIRE-qPCR at the regions used in the screen in EWS894 cells treated with MS0621 or a control compound for 16 (h) Results are shown as a fraction of input control. Error bars represent the standard error of four biological replicates for MS0621 and two replicates for the control compound. Statistical significance compared to the lowest concentration of each compound was assessed using unpaired student t-tests *p < 0.05, **p < 0.01, ns: not significant. **(D)** FAIRE-qPCR at EWSR1::FLI1-bound and control loci in EWS894 cells treated with 10 μM MS0621 or vehicle control (DMSO) for 16 hours. Results are shown as a fraction of input control normalized to vehicle control. Error bars represent the standard error of three biological replicates. Statistical significance compared to the vehicle control at each locus was assessed using unpaired student t-tests *p < 0.05, **p < 0.01, ***p < 0.001, ****p < 0.0001 ns: not significant. **(E)** FAIRE-qPCR in the indicated Ewing sarcoma cell lines treated with 5 μM MS0621 or vehicle control (PBS) for 16 (h) Results are shown as Log2 RCI scores. Dashed lines indicate –2 SDs from the average RCI for vehicle controls. Error bars represent the standard deviation of two biological replicates. **(F)** ChIP-qPCR at EWS-FLI-bound (P1, P7, CAV2) and control regions (Neg) in A673 cells treated with 5 μM MS0621 or vehicle control (PBS) for 16 (h) Results are shown as a fraction of input control. Error bars represent the standard deviation of two biological replicates.

We previously performed a high-throughput, small molecule, chromatin-based (FAIRE-qPCR) screen of a curated set of chromatin-targeted small molecules (9). Molecules were scored based on their ability to affect FAIRE signal at two FAIRE-enriched, EWSR1::FLI1-bound sites in Ewing sarcoma relative to regions of FAIRE signal shared by multiple cell types. The relative FAIRE signal difference (Relative Chromatin Inhibition, RCI) for each compound was calculated as previously described (9). Fifty-eight compounds demonstrated RCI scores lower than 2 standard deviations from the scores of vehicle-treated control cells (**Figure 1A**, **Supplemental Table 1**). Among the previously uncharacterized hit compounds, we identified UNC0621, which has since been renamed MS0621 (**Figure 1B**). To confirm the activity of MS0621, we performed FAIRE-qPCR at the screening regions on EWS894 cells treated with several concentrations of MS0621 or a control compound which did not score as a hit in the initial screen (**Figure 1C**). We found that MS0621 decreased FAIRE signal at the screening sites in a concentration dependent manner.

We then evaluated the effect of MS0621 on FAIRE signal at regions beyond those in the screen. MS0621 decreased FAIRE signal at seven FAIRE-positive, EWSR1::FLI1 bound regions, including additional sites associated with EWSR1::FLI1 regulated genes (**Figure 1D**). We then tested the activity of MS0621 in two additional Ewing sarcoma cell lines (EWS502 and A673). As in EWS894 cells, MS0621 decreased FAIRE signal at the screening loci in both cell lines (**Figure 1E**). Previously, we demonstrated that HDAC inhibitors affect Ewing satrcoma cell chromatin by downregulating EWSR1::FLI1 protein levels. To ask whether MS0621 also affected EWSR1::FLI1 levels, we assessed EWSR1::FLI1 RNA and protein following MS0621 treatment (**Supplemental Figures 1A, B**). MS0621 does not decrease either EWS-FLI expression or protein abundance. Although RNA abundance may increase, protein levels remained constant. We then asked whether MS0621 displaces EWSR1::FLI1 from chromatin. MS0621 treatment did not affect EWSR1::FLI1 binding to chromatin assessed by chromatin immunoprecipitation (ChIP-qPCR) (**Figure 1F**). Taken together, we identified MS0621 as a small molecule that attenuates FAIRE signal at EWSR1::FLI1-bound loci through a mechanism independent of altered EWSR1::FLI1 levels or chromatin binding.

### MS0621 induces a G1/S cell cycle arrest

**Figure 2:**
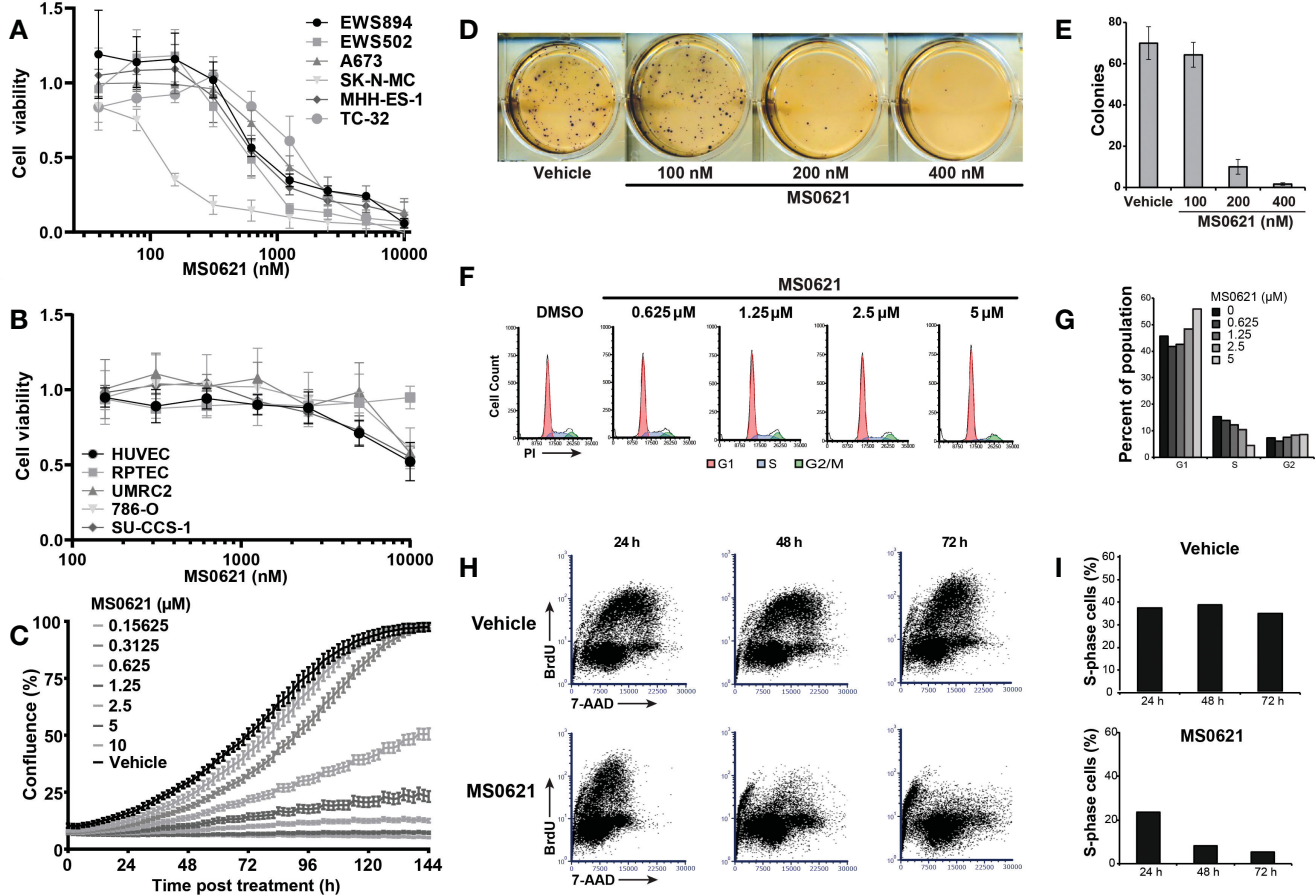
MS0621 induces a G1/S arrest in Ewing sarcoma cell lines. **(A)** Cell proliferation was assayed following 3 days of treatment with MS0621 or a vehicle control (PBS) in the indicated Ewing sarcoma cell lines. Viable cells were assessed by WST-1. Results are shown as the fold change relative to vehicle treated cells. Error bars represent the SD of three biological replicates. **(B)** Cell proliferation in UMRC2 (clear cell renal cell carcinoma, ccRCC), 786-O (ccRCC), RPTEC (Primary Renal Proximal Tubule Epithelial Cells), HUVEC (Primary Umbilical Vein Endothelial Cells), and SU-CCS-1 (clear cell sarcoma) cells treated with MS0621 or vehicle control for 3 days. Proliferation was assessed on day 3 by WST. Results are shown as the fold change of vehicle treated cells. Error bars represent the SD of three biological replicates. **(C)** Cell proliferation was assessed by live cell imaging in A673 cells treated with MS0621 or vehicle control over 6 days. Concentrations are in micromolar. Proliferation, assessed by images captured every 2 hours, are shown as the percent confluence. Error bars represent the standard error of the mean for six (MS0621 treatment) technical replicates or two (vehicle control) technical replicates. **(D)** Soft agar colony formation assays for EWS894 cells treated with the indicated doses of MS0621 or vehicle (DMSO) for 15 days. Viable cell colonies were visualized on day 15 with MTT. Results shown are representative wells of three biological replicates. **(E)** Quantification of soft agar colony formation. Error bars represent the SD of three biological replicates. **(F)** Cell cycle analysis in EWS502 cells treated with the indicated doses of MS0621. Following treatment with MS0621 or vehicle (DMSO) for 3 days, cells were stained with propidium iodide (PI) and analyzed by flow cytometry. **(G)** Quantification of the percent of the population in each stage of the cell cycle for each treatment condition in **(F)**. **(H)** S-phase quantification by BrdU staining in EWS502 cells treated with 5 μM MS0621or vehicle (DMSO). Following compound treatment for the indicated time, cells were pulsed with BrdU for 1 hour, harvested, fixed, and analyzed by flow cytometry. **(I)** Quantification of the BrdU-positive population in **(H)**. Results are shown as the percent of cells in S-phase.

**Figure 3:**
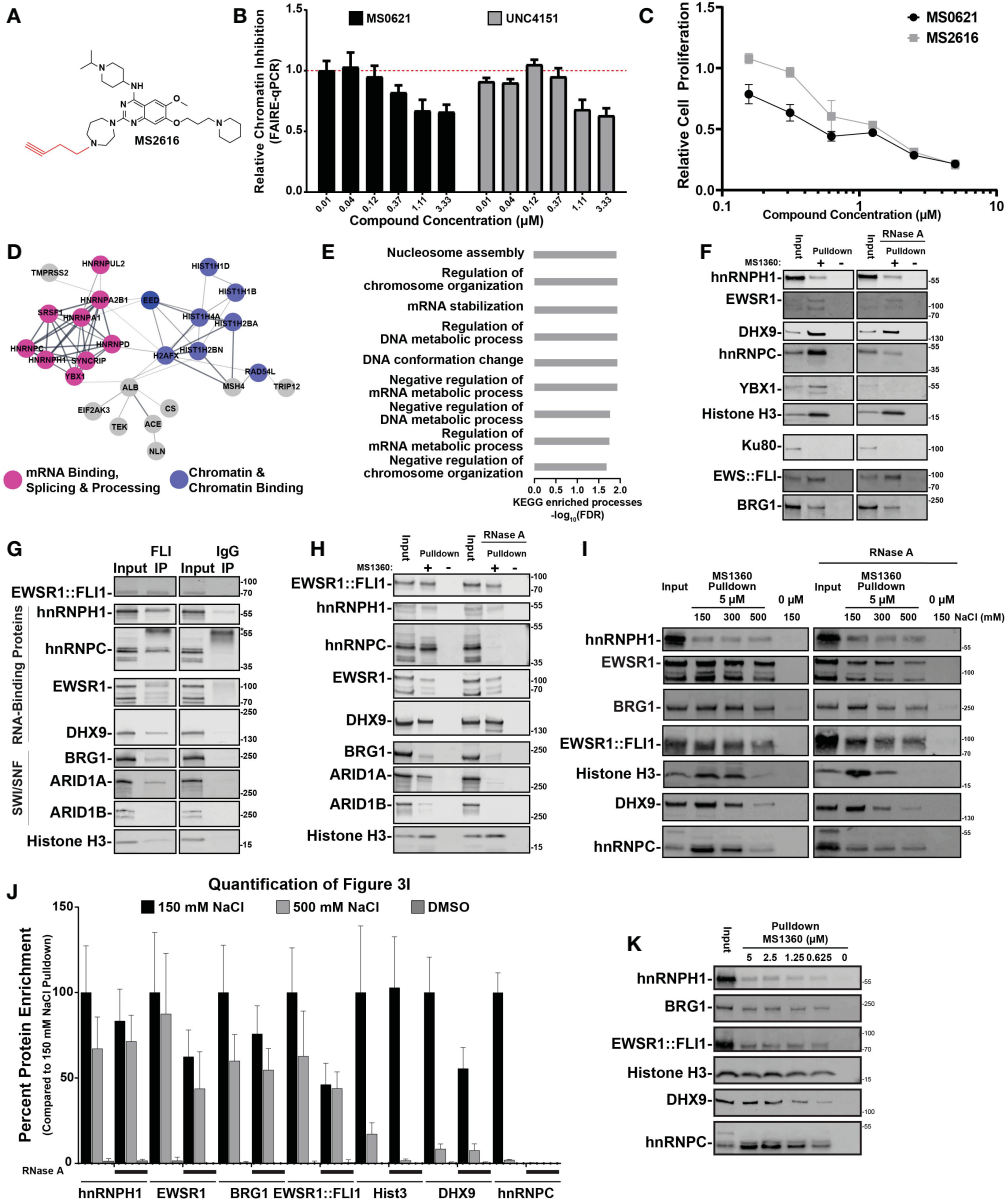
MS0621 active analog interacts with a macromolecular complex enriched with RNA-binding proteins **(A)** Chemical structure of MS2616, alkyne-substituted analog of MS0621. **(B)** FAIRE-qPCR at the screening loci in EWS894 cells treated with the indicated doses of MS0621 or UNC4151 for 16 hours. Results are shown as Relative Chromatin Inhibition. Error bars represent the standard deviation of 5 technical replicates. **(C)** Cell proliferation curves for EWS894 cells treated with the indicated doses of MS0621 or MS2616 (twofold dilutions from 5 μM to 0.15625 μM) or vehicle control for 3 days. Proliferation was assessed on day 3 by WST assay. Results are shown as the fold change of vehicle treated cells. Error bars represent the SD of three biological replicates. **(D)** STRING diagram displaying interactions networks between proteins identified by mass spectrometry in EWS894 cells. **(E)** KEGG Enriched Biological Processes GO terms for proteins identified by mass spectrometry in EWS894 cells. Results shown are the -log_10_ FDR for the top 50% of enriched GO terms. **(F)** Western blot confirmation of proteins identified by mass spectrometry. Proteins chemiprecipitated by 5 μM MS1360 (+) or vehicle control (–) in MNase-digested nuclear extracts of EWS894 cells with or without 100 ug/mL RNase A digestion assessed by western blot. 20% of the MNase-digested nuclear extract input was included for reference (Input). **(G)** Western blot analyses of proteins immunoprecipitated by EWSR1::FLI1 from nuclear extracts of A673 cells. 6% of the input extract was included for reference. Antibody heavy chain is marked with an asterisk. **(H)** Western blot analyses of proteins enriched by 5 μM MS1360 (+) or vehicle control (–) in nuclear extracts of A673 cells with or without 100 ug/mL RNase A digestion assessed by western blot. 10% of the nuclear extract input was included for reference. **(I)** MNase-digested extracts of A673 cells were chemiprecipitated with MS1360 in the presence 150 mM NaCl. Beads were then sequentially washed with the indicated concentrations of NaCl. Beads were removed at each condition for Western blot analyses. 20% of the MNase-digested nuclear extract input was included for reference. **(J)** Quantification of western blot band intensities for protein enrichment under standard (150 mM NaCl) and high salt (500 mM NaCl) conditions normalized to Input band intensities. Addition of RNase A is indicated by black lines beneath the bars. Results are shown as the fold change of the 150 mM NaCl condition. Error bars represent the standard deviation of three technical replicates. Hist3: Histone H3. **(K)** Western blot analyses of chemipreciptation of MNase-digested extracts of A673 cells in the presence of decreasing concentrations of MS1360. 20% of the MNase-digested nuclear extract input was included for reference.

We next asked whether MS0621 affected cell proliferation. Several Ewing sarcoma cell lines were treated with MS0621 for three days after which viability was assessed (**Figure 2A**). MS0621 resulted in a concentration-dependent decrease in cell proliferation in all tested Ewing sarcoma cell lines. Most cell lines had an IC50 of less than 1 μM (**Table 4**). One cell line (SK-N-MC) exhibited exquisite sensitivity with an IC50 of 128.6 nM (95% confidence interval 94.49-153.5 nM). To assess whether the anti-proliferative effect of MS0621 demonstrated cell type specificity, we assayed several other cell lines. Among these were epithelial carcinoma cell lines (UMRC2 and 786-O), a clear cell sarcoma cell line with the oncogenic EWS::ATF1 rearrangement (SU-CCS-1), and two primary cell lines that either express FLI1 (HUVEC) or do not express FLI1 (RPTEC) (**Figure 3B**). MS0621 did not significantly inhibit the proliferation of any of these cells at the tested concentrations. Assessment of proliferation using live cell imaging in A673 and MHH-ES cells demonstrated that MS0621 rapidly and persistently inhibited Ewing sarcoma cell proliferation (**Figure 3C**; **Supplemental Figure 2A**). Proliferation defects were apparent within hours of MS0621 treatment and persisted over the course of the experiment. We then evaluated the effect of MS0621 in the context of anchorage-independent growth (**Figures 3D, E**). MS0621 reduced EWS894 colony formation in soft agar assays at sub-micromolar concentrations.

**Table 4:** MS0621 IC_50_ Values in Ewing sarcoma Cell Lines.

Cell Line	IC_50_ (nM)	95% Confidence Interval
SK-N-MC	128.6	94.49 - 153.5
EWS502	532.1	401.3 - 716.8
EWS894	557	420.2 - 790.8
MHH-ES-1	559.6	437.3 - 729.7
A673	891.2	728.8 - 1116
TC-32	1511	1229 - 1901

We next explored the mechanisms underlying decreased proliferation. By Annexin-V staining and flow cytometry, we found that MS0621 did not induce apoptosis (**Supplemental Figure 2B**). To test the effect of MS0621 on cell division, Ewing sarcoma cells were stained with a cell permeable, covalent binding fluorescent dye (CFSE, **Supplemental Figure 2C**). As expected, CFSE staining intensity decreased over time in untreated cells due to cell proliferation. However, in MS0621-treated cells, following an initial decrease, signal remained constant indicating the absence of cell division.

We next explored the specific cell cycle effects of MS0621. EWS502 cells treated with MS0621 for 72 hours were stained with propidium iodide (PI) and analyzed by flow cytometry (**Figures 3F, G**). Increased concentrations of MS0621 were associated with a near complete loss of cells in S-phase and an increased fraction of cells in G1. This effect was apparent by 48 hours of treatment, in support of the CFSE results (**Supplemental Figures 2C, D**). To specifically quantify cells in S phase, we performed BrdU labeling (**Figures 3H, I**). Compared to vehicle, MS0621 treatment decreased the fraction of cells in S-phase. While appreciable at 24 hours, the magnitude of this effect increased at 48 and 72 hours. Taken together, these data suggest that MS0621 decreases proliferation, including anchorage-independent growth, in Ewing sarcoma cells by inducing a profound and sustained cell cycle arrest.

### MS0621 interacts with an RNA-binding protein containing macromolecular complex

MS0621 was synthesized as part of a quinazoline scaffold diversity series generated to increase the *in vitro* potency of UNC0321, a small molecule G9a inhibitor. In addition to MS0621, several compounds with putative G9a activity were identified as hits in the initial chromatin-based screen, including other analogs from the diversity series (**Supplemental Figure 3A**). We then asked whether these analogs affected cell proliferation. Neither the other analogs in the series, UNC0618 and UNC0559, nor other putative G9a inhibitors, UNC0127 and UNC0528, exhibited a similar IC50 as MS0621, although UNC0618 and UNC0559 demonstrated activity at higher concentrations (**Supplemental Figure 3B**). These data suggest that effects on chromatin state do not fully correlate with cell cycle effects. Because MS0621 was generated as part of a G9a inhibitor series, we asked whether MS0621 affected H3K9 methylation in Ewing sarcoma cells. Whereas UNC0638 potently decreased H3K9me2, MS0621 slightly increased H3K9me2 (**Supplemental Figure 3C)**. Additionally, neither UNC0638 nor its analogs, UNC0642 and UNC0737, exhibited similar antiproliferative effects on Ewing sarcoma cell lines as MS0621 (**Supplemental Figure 3D**). Furthermore, UNC0638 did not score as a hit in the initial chromatin-based screen (**Supplemental Figure 3A**). These data suggest that MS0621 does not mediate its effects on Ewing sarcoma cells through a mechanism dependent on G9a inhibition. Furthermore, these data point to a mechanism distinct from other quinazoline scaffold derivatives identified in the screen.

In the absence of a predicted target, we next sought to identify the protein interactors of MS0621. We generated an alkene (UNC4151) and alkyne (MS2616)-substituted analogs of MS0621, which could be used for the generation of affinity reagents (**Figure 3A**). We then assessed whether the alkyne addition affected the molecular and cellular activities of MS0621. UNC4151 decreased FAIRE signal at EWSR1::FLI1-bound loci and both MS2616 and UNC4151 decreased cell viability in Ewing sarcoma cells in a concentration-dependent manner similar to MS0621 (**Figures 2B, C**; **Supplemental Figure 3E**). We then used Copper-Catalyzed Azide-Alkyne Cycloaddition click chemistry to generate MS1360, a biotinylated analog of MS2616 as an affinity reagent.

As MS0621 altered chromatin state, we hypothesized that relevant interactions would be with chromatin-bound proteins. To preserve native chromatin-dependent interactions, we generated extracts from micrococcal nuclease (MNase)-digested EWS894 and HEK293T nuclei. Extracts were incubated with MS1360 and then bound to streptavidin-conjugated beads. Isolated complexes were then subjected to tryptic digestion and mass spectrometry. STRING network analysis of proteins identified by mass spectrometry indicated that MS1360 interactors were enriched for RNA and DNA binding proteins in both cell lines (**Figures 2D, E**; **Supplemental Figures 3F, G**, **Supplemental Table 2**, **3**). Identified proteins were enriched in biological processes involved in chromatin regulation and RNA processing, including multiple HNRNPs and histone proteins. We next sought to confirm these interactions. Numerous RNA binding proteins such as hnRNPH1, hnRNPC, YBX1, and EWSR1 were pulled down by MS360. However, the abundant nuclear DNA damage protein Ku80 was not detected, indicating selectivity of MS0621 with nuclear interactors (**Figure 2F**, **Supplemental Figure 3H**). Interactions with these proteins were also observed in additional Ewing sarcoma cell lines (A673 and EWS502, **Supplemental Figure 3I**). We also tested these interactions in SU-CCS-1 cells, as they harbor an EWSR1::ATF1 rearrangement, and 786-O, an epithelial renal carcinoma cell line. Similar interactions were identified suggesting that MS1360 interacts with an overlapping set of proteins across cell types (**Supplemental Figure 3I**). Interestingly, although MS1360 pulled down both the EWSR1::ATF1 fusion protein and the parental ATF1 transcription factor in SU-CCS-1 cells, ATF1, which was present in the other cell lines, was not pulled down. The inclusion of ATF1 in the MS1360-interacting complex in SU-CCS-1 cells may reflect the contribution of the EWSR1 domain to the fusion (20). These data demonstrate that MS0621 interacts with a complex enriched with proteins involved in transcription and co-transcriptional processes.

Since the chromatin extract retained RNA, and RNA-associated proteins were detected by mass spectrometry, we explored the effect of RNA on protein interactions. Treatment of the extracts with RNase A during the MS1360 pulldown did not affect interactions with hnRNPH1, EWSR1, or Histone H3 but attenuated interactions with DHX9, hnRNPC, and YBX1. (**Figure 2F**; **Supplemental Figure 3H**). As these RNase A-sensitive proteins interact with R-loops, we next explored whether these interactions were disrupted by digesting the RNA moiety of RNA-DNA hybrids (21–23). In contrast to RNaseA, digestion with RNaseH did not alter the interaction of these proteins with MS1360 (**Supplemental Figure 3J**). These results suggest that MS0621 interacts with a macromolecular complex enriched in RNA interacting proteins, but independent of RNA.

Because several of the MS1360-interacting proteins were also known or putative interactors of EWSR1::FLI1, we assessed whether MS1360 also interacts with EWSR1::FLI1. We found that in Ewing sarcoma cells, MS1360 interacts with EWSR1::FLI1 (**Figure 2F**; **Supplemental Figure 3J**). We next asked whether EWSR1::FLI1 interacts with the MS1360-interacting proteins hnRNPH1 and hnRNPC. We generated nuclear extracts by MNase digestion or by hypotonic cellular lysis and mechanical shearing. EWSR1::FLI1 co-precipitated with hnRNPH1 and hnRNPC in both extracts as did known EWSR1::FLI1-interacting RNA binding proteins DHX9 and EWSR1 (**Figure 2G**; **Supplemental Figure 3K**). As with MS1360, the interaction between EWSR1::FLI1 and hnRNPC, but not hnRNPH1, was attenuated with RNase A digestion. These data indicate that EWSR1::FLI1 also interacts with MS0621-interacting proteins in Ewing sarcoma cells.

EWSR1 and EWSR1::FLI1 are known to cooperate to recruit the SWI/SNF complex to EWSR1::FLI1-bound loci, and SWI/SNF components have been implicated in Ewing sarcoma (7). Consistent with published results, we observed that EWSR1::FLI1 co-immunoprecipitated the SWI/SNF components BRG1 and ARID1A, but not ARID1B (**Figure 2G**; **Supplemental Figure 3K**) (7, 24). In contrast, MS1360 interacts with both ARID1A and ARID1B as well as BRG1(**Figure 2H**). We also observed that MS1360 interacted with BRG1 in HEK293T cells, suggesting that SWI/SNF components interact with MS1360 independently of EWSR1::FLI1 (**Supplemental Figure 3H**). ARID1A-containing SWI/SNF complexes are associated with actively transcribed regions whereas ARID1B-containing SWI/SNF is associated with repressed enhancers (25, 26). The interaction of EWSR1::FLI1 with ARID1A-, but not ARID1B-contatining SWI/SNF is consistent with EWSR1::FLI1’s association with enhancers and actively transcribed regions. These data suggest that MS0621 interacts with a broader range of protein complexes, including those at both active and repressed chromatin.

To more narrowly define the core MS0621-interacting complex, we explored the affinity of MS1360-protein interactions. We noted that the interactions of some proteins appeared to be attenuated in those extracts produced without enzymatic digestion (**Figures 2H, I**). We hypothesized that the higher NaCl compared to MNase-digested extracts destabilized the interaction with select interactors. Using increasing NaCl concentrations, we sought to identify high affinity MS1360-interacting proteins. We observed that interactions with histone H3, DHX9 and hnRNPC were attenuated in the presence of increasing concentrations of NaCl (**Figures 2I, J**; **Supplemental Figure 3L**). This effect was even more pronounced in the RNase A treated chromatin. In contrast, interactions with hnRNPH1, EWSR1, BRG1, and EWSR1::FLI1 were more resistant to both high NaCl and RNaseA, with over 50% of hnRNPH1 enrichment maintained at 500 mM NaCl with or without RNase A. Interestingly, we observed that the interaction with histone H3 was almost completely lost in the presence of high NaCl and RNaseA, which is consistent with decreased nucleosome stability at this concentration (27–29). These data suggest that while the interactions of MS1360 with the NaCl resistant proteins appear to take place on chromatin, the interactions are not chromatin dependent. As MS0621 exerted effects on chromatin and cell proliferation at nanomolar concentrations, we next asked whether these interactions were maintained at similar concentrations of biotinylated-MS1360. We found that the enrichment of proteins was attenuated with decreasing concentrations of MS1360 but persisted to the lowest concentration tested, 625 nM (**Figure 2K**). These results suggest that MS0621 interacts with a core complex of proteins that includes hnRNPH1, EWSR1, BRG1, and EWSR1::FLI1 in the absence of RNA or intact chromatin.

### hnRNPH1 knockdown phenocopies MS0621 in Ewing sarcoma cells

**Figure 4:**
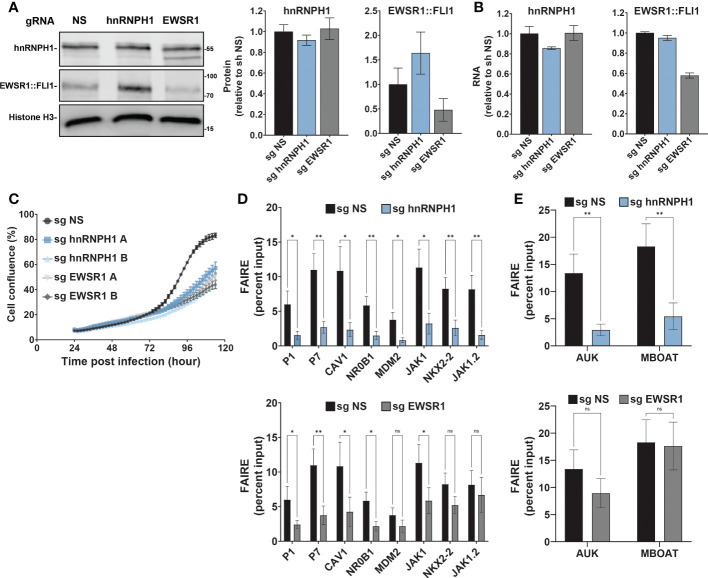
hnRNPH1 knockdown recapitulates the effect of MS0621 on Ewing sarcoma cell proliferation and FAIRE-qPCR **(A)** Western blot analyses of hnRNPH1 and EWSR1::FLI1 protein knockdown in A673-CRISPRi cells. Left: Representative western blots. Right: Quantification of western blot band intensities for hnRNPH1 and EWSR1::FLI1 normalized to Histone H3 band intensities. Results are shown as the fold change of non-specific guide (NS) cells. Error bars represent the standard deviation of three biological replicates. **(B)** hnRNPH1 and EWSR1::FLI1 RNA abundance measured by RT-qPCR in A673-CRISPRi cells following infection with the indicated sgRNAs for 4 days. Results are shown as the fold change of non-specific guide (sg NS) cells. Error bars represent the standard deviation of three biological replicates. **(C)** Cell proliferation assayed by live cell imaging for A673-CRISPRi cells infected with the indicated sgRNAs over 4 days. Images were obtained every 2 hours. Results are shown as the percent confluence. Error bars represent the standard error of the mean for two technical replicates where each replicate consists of 16 images per time point. Two biological replicates **(A, B)** are shown for hnRNPH1 and EWSR1. Nonspecific sgRNA was used as a control (NS). **(D)** FAIRE-qPCR in A673-CRISPRi cells infected with lentivirus transducing sgRNA for hnRNPH1 for 4 days (Left: EWSR1::FLI1 target regions. Right: Positive control regions). Results are shown as a fraction of input control. Error bars represent the standard error of three biological replicates. **(E)** FAIRE-qPCR in A673-CRISPRi cells infected with lentivirus transducing sgRNA for EWSR1 for 4 days (Left: EWSR1::FLI1 target regions. Right: Positive control regions). Results are shown as a fraction of input control. Error bars represent the standard error of three biological replicates. Control sgRNA results are duplicated in Figures **(D, E)**. The asterisks indicate the significance of unpaired t-tests where *p < 0.05 and **p < 0.01. ns, nonsignificant.

As hnRNPH1 interacted robustly with MS1360 under all tested conditions, we hypothesized that hnRNPH1 is a core member of the MS0621-interacting complex and that loss of hnRNPH1 would recapitulate the effects of MS0621 on Ewing sarcoma cells. To test this hypothesis, we used CRISPRi-mediated silencing of hnRNPH1 and EWSR1 in Ewing sarcoma cell lines. We confirmed that the EWSR1-targeting sgRNA decreased EWSR1::FLI1 protein and RNA levels similarly to an shRNA directed to the 3′ UTR of FLI1 (shFLI) (**Figures 4A, B**, and **Supplemental Figures 4A**, **B**) (6). EWSR1::FLI1 CRISPRi knockdown had the same effect on FAIRE signal at EWSR1::FLI1-bound loci as lentiviral mediated EWSR1::FLI1 silencing (**Supplemental Figure 4C**).

Although the effect of the hnRNPH1 silencing seemed modest, EWSR1::FLI1 levels increased and Ewing sarcoma cell line proliferation was dramatically reduced (**Figure 4C**; **Supplemental Figure 4D**). Compensation in loading to account for cell number differences may partially obscure the reduction in expression. Silencing of hnRNPH1 decreased FAIRE signal at EWSR1::FLI1-bound loci, similar to that observed for EWSR1::FLI1 knockdown (**Figures 4D, E**; **Supplemental Figure 4E**). Interestingly, in contrast to EWSR1::FLI1 loss which did not affect FAIRE signal at non-GGAA repeat-containing EWSR1::FLI1-bound loci (NKX2-2 and JAK1.2), hnRNPH1 knockdown, like MS0621, decreased FAIRE signal at all tested EWSR1::FLI1-bound loci (**Figure 1D**, **Figures 6D, E**). Silencing hnRNPH1 also decreased FAIRE signal at the positive control loci, in contrast to MS0621 treatment and EWSR1::FLI1 knockdown (**Figures 4D, E**; **Supplemental Figure 4F**). These results suggest that attenuation of hnRNPH1 has broader effects on chromatin states in Ewing sarcoma than MS0621 treatment. The striking effects associated with modest decreases in hnRNPH1 levels may reflect the toxicity of hnNRPH1 loss in those cells with the earliest or most effective silencing of hnRNPH1. Taken together, these data suggest that loss of hnRNPH1 partially phenocopies the effects of MS0621 on EWSR1::FLI1-dependent phenotypes in Ewing sarcoma. These data also suggest that perturbation of the RNA processing machinery through loss of hnRNPH1 is a sensitivity of Ewing sarcoma cells.

### MS0621 affects EWSR1::FLI1-mediated gene expression and alternative splicing

**Figure 5:**
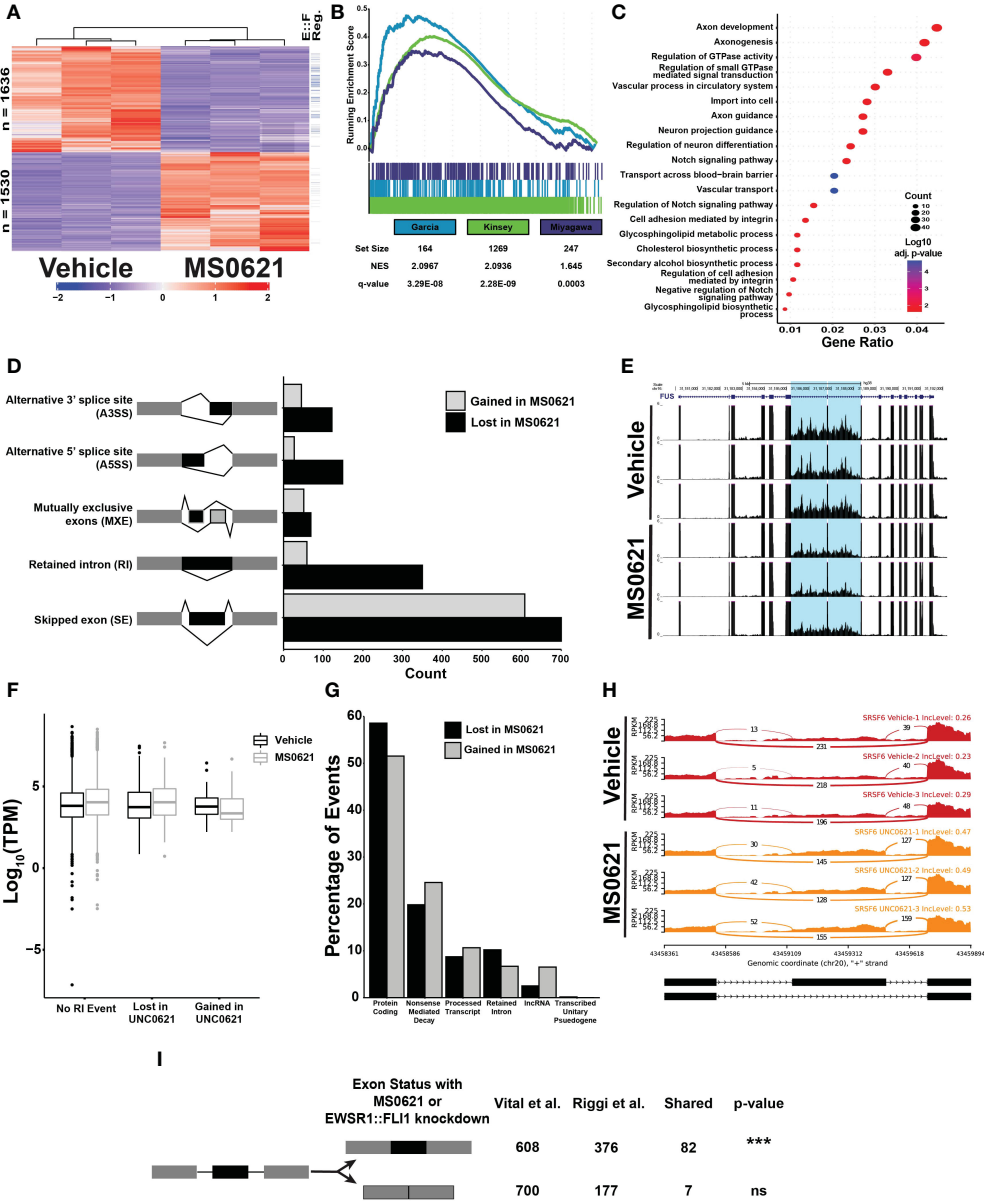
MS0621 affects EWSR1::FLI1 regulated genes and influences RNA splicing **(A)** Heatmap displaying row normalized Z-scores for differentially expressed genes in EWS894 cells following 16 hours of treatment with 5 μM MS0621 or vehicle control (DMSO). The right most column indicates genes also differentially regulated by EWSR1::FLI1 in (30). **(B)** Gene set enrichment analysis of genes differentially expressed in EWS894 cells following treatment for 16 hours with 5 μM MS0621 or vehicle control (DMSO) using gene sets upregulated by EWSR1::FLI1. **(C)** Enriched Biological Processes GO terms for genes that were downregulated with MS0621 treatment but not regulated by EWSR1::FLI1. Results shown are the -log_10_ adjusted p-value for the top 20 enriched GO terms. **(D)** Significant differential alternative splicing events were identified by rMATS from RNA prepared from EWS894 cells treated 16 hours with 5 μM MS0621 or vehicle control (DMSO). Indicated splicing events were supported by at least 20 reads, an Inclusion Level Difference > 10%, and FDR < 0.05. **(E)** Differential retention of a tumor-characteristic pair of introns in the FUS gene. Gene tracks represent three replicates each for Vehicle- or MS0621- treated EWS894 cells. **(F)** Box plots of log10 TPM values for genes with an RI event gained or lost with MS0621 or with no RI event. **(G)** Percentage of transcript-specific SE events identified by rMATS annotated by GENCODE biotype. **(H)** Sashimi plots of differential retention of the SRSF6 poison exon. Sashimi plots represent three replicates each for Vehicle- or MS0621- treated EWS894 cells. **(I)** Enrichment of SE events gained with MS0621 and EWSR1::FLI1 was assessed by permuting 1,000 times over spliceable exons with minimum read coverage greater than or equal to 20 reads. ***p-value less than 0.001.

As many MS0621 interactors are involved in RNA processing, we next asked whether MS0621 alters transcription in Ewing sarcoma cells. We identified differentially expressed genes using RNA-seq in EWS502 cells treated with MS0621 for 16 h. Slightly more genes were downregulated (1636) than upregulated (1530) with MS0621 treatment (**Figure 5A**; **Supplemental Table 4**). We tested whether there is an association between genes differentially regulated by MS0621 treatment and those regulated by EWSR1::FLI1. Using Gene Set Enrichment Analysis (GSEA), we found genes downregulated with loss of EWSR1::FLI1 were enriched among genes downregulated with MS0621 treatment (**Figures 5A, B**; **Supplemental Table 5**) (30–32). Genes downregulated by MS0621 and EWSR1::FLI1 knockdown were enriched for biological pathways related to DNA damage response and cell cycle regulation (**Supplemental Figures 5A, B**) (30). The shared regulation of this class of genes may reflect cell cycle arrest.

We next explored the effects of MS0621 on gene expression more generally. Genes upregulated by MS0621 were enriched for GO Terms related to cilium- and microtubule-based organization and movement (**Supplemental Figure 5D**, **Supplemental Table 5**). Genes downregulated by MS0621 were also enriched for DNA damage repair and cell cycle regulation (**Supplemental Figure 5C**, **Supplemental Table 5**). To explore other pathways downregulated by MS0621, we evaluated genes that were regulated by MS0621 but not EWSR1::FLI1 loss. Genes specifically downregulated by MS0621 were enriched for neuronal development and differentiation, GTPase signaling, and Notch signaling (**Figure 5C**). Interestingly, GTPase and Notch signaling have been implicated in regulating neuronal differentiation and SWI/SNF activity in Ewing sarcoma (33–35). These data indicate that MS0621 alters the expression of genes regulated by EWSR1::FLI1 and genes implicated in Ewing sarcoma biology. Among the genes most strongly downregulated by MS0621 are MYBL2 and RRM2. MYBL2 has been identified as a direct target of EWSR1::FLI1 with germline polymorphisms associated with activation in Ewing sarcoma and RRM2 overexpression is associated with poor prognosis Ewing sarcoma (36, 37).

As MS0621 interacts with many RNA binding and processing proteins, we next asked whether MS0621 alters splicing. Splicing analysis (rMATS) identified 2180 alternative splicing (AS) events (**Figure 5D**; **Supplemental Table 5**) (18). In comparison, knockdown of EWSR1::FLI1 or the splicing regulator RBFOX2 resulted in 1880 and 768 alternative splicing events, respectively. This indicates that MS0621 affects splicing to a similar degree as perturbing proteins known to regulate splicing in Ewing sarcoma (38). The most abundant classes of AS events were Skipped Exon (SE) and retained introns (RI) (1308, 60% and 409,18.8%, respectively). More than five times as many RI events were lost with MS0621 treatment (350) as were gained (59). As increased intron retention is characteristic of tumors compared to normal tissues, we next sought to characterize the differential RI events (39, 40). Analysis of cancer-associated splicing in TCGA tumors found that RNA-splicing factors were enriched among introns retained in multiple cancers (39). Similarly, genes with RI events that were lost with MS0621 treatment were enriched for those involved in RNA processing (**Supplemental Figure 5E**, **Supplemental Table 7**). For example, FUS has two adjacent introns which are commonly retained in tumors (39). These introns were lost following MS0621 treatment (**Figure 5E**). Although there was enrichment of RNA splicing, miRNA processing, and protein signaling genes among those with RI events gained with MS0621 treatment, they these did not meet statistical significance (**Supplemental Table 7**).

We next assessed whether intron retention was associated with the expression of the involved genes (**Figure 5F**). RNA abundance was slightly higher in MS0621 treated cells compared to vehicle treated controls for both genes lacking RI events and those for which RI events were lost with MS0621.In contrast, the expression of genes that gain RI events with MS0621 was decreased in MS0621 treated cells compared to vehicle treated controls. Consistent with previous reports, these data support a role for intron retention in decreasing the abundance of associated transcripts (39, 41). These data further indicate that MS0621 modulates intron retention to influence gene expression.

We next explored whether MS0621 alters gene expression through SE events, the most abundant class of AS events. Alternative splicing coupled to nonsense-mediated mRNA decay (AS-NMD) is a conserved mechanism by which the expression of many genes is regulated (42–44). Given the enrichment of splicing factors among RI events and the association of intron retention with NMD, we hypothesized that MS0621 could dysregulate AS-NMD (45, 46). We identified 1258 SE events in transcripts, slightly fewer than with gene-level analysis (1308). The detection of more events in genes compared to transcripts likely reflects the decreased statistical power to call SE events in individual transcripts relative to the multiple transcripts combined for gene-level analysis. Slightly more SE events were lost (676, 53.7%) than gained (582, 46.3%) with MS0621. Protein Coding and Nonsense-mediated decay were the two most abundant annotated biotypes (696, 55.3% and 277, 22.0%, respectively) (**Figure 5G**; **Supplemental Table 9**).

Among SE events lost with MS0621 treatment, approximately 20% (134) were in transcripts projected to undergo nonsense-mediated decay. The associated genes were enriched in biological processes involved in RNA splicing and microRNA processing (**Supplemental Figure 5F**; **Supplemental Table 9**). These data are consistent with the enrichment of GO Terms associated with RNA processing in RI events lost with MS0621 treatment. Nearly 25% (143) of SE events gained with MS0621 treatment were in transcripts projected to undergo nonsense-mediated decay. GO Terms associated with these events included RNA splicing and macromolecule methylation though these did not meet statistical significance for enrichment (**Supplemental Table 9**). Many transcripts that undergo AS-NMD include ultraconserved genomic loci, regions that are perfectly conserved in the human, mouse, and rat genomes (47–49). The association of highly conserved regions and splicing regulation is conserved among metazoa, indicating the importance of these regions in regulating splicing (47, 50). We explored whether these transcripts were present among those altered by MS0621 and observed eight SE events in genes with ultraconserved elements, seven of which were gained with MS0621. Most genes with events gained with MS0621 have known functions in AS, including 4 of 14 members of the SR family of splicing factors (SRSF6, SRSF11, TRA2A, and TRA2B), hnRNPDL, and OGT (**Figure 5H**) (49, 51). These data indicate that MS0621 shifts patterns of AS-NMD.

As EWSR1::FLI1 has been implicated in regulating alternative splicing (24, 38, 52–57), we next explored whether SE events observed with MS0621 treatment were shared with those regulated by EWSR1::FLI1. Splicing analysis of published data in Ewing sarcoma cells with and without EWSR1::FLI1 identified 921 AS events (**Supplemental Figure 5G**) (8). As with MS0621, SE events were the most abundant class of AS (553, 60%). SE events gained with EWSR1::FLI1 knockdown were significantly enriched those gained with MS0621 treatment (p-value < 0.001, **Figure 5I**). Events shared between the two data sets included those associated with ultraconserved AS-NMD events such as TRA2A and SRSF6. In contrast to MS0621 treatment, EWSR1::FLI1 knockdown increased the number of SE events (376, 68%) compared to control (177, 32%). This relationship of increased SE with EWSR1::FLI1 knockdown (781, 57.4%) than in control (579, 42.6%) was also observed in an independent data set (38). These data indicate that MS0621 reprograms important alternative splicing patterns, many of which are also regulated by EWSR1::FLI1.

## Discussion

In this report we describe the biochemical interactors and molecular consequences of treatment with MS0621, a small molecule identified in a screen of compounds that reverse EWSR1::FLI1-mediated chromatin accessibility. We found that MS0621 treatment recapitulated the effects of EWSR1::FLI1 loss on FAIRE, cell cycle, and gene expression through a mechanism independent of regulating EWSR1::FLI1 gene expression. We identified a range of protein interactors of MS0621, which included EWSR1::FLI1 and several proteins involved in RNA splicing and chromatin state regulation.

Though MS0621 shares structural similarity with the G9a inhibitor UNC0638, it did not alter H3K9me2 levels in the Ewing sarcoma cells tested. Further, G9a inhibitors did not phenocopy the effect of MS0621 on either chromatin accessibility or cell proliferation. Differences in G9a inhibitory activity between the two compounds may be attributable to substitution of the 7-(3-pyrrolidin-1-yl-) propoxy side chain by which UNC0638 interacts with the G9a lysine binding channel with a bulkier 1,4-diazepin-1-yl side chain in MS0621 (58). This chemical substitution may also contribute the interactions we observed between MS0621 and nuclear proteins.

**Figure 6:**
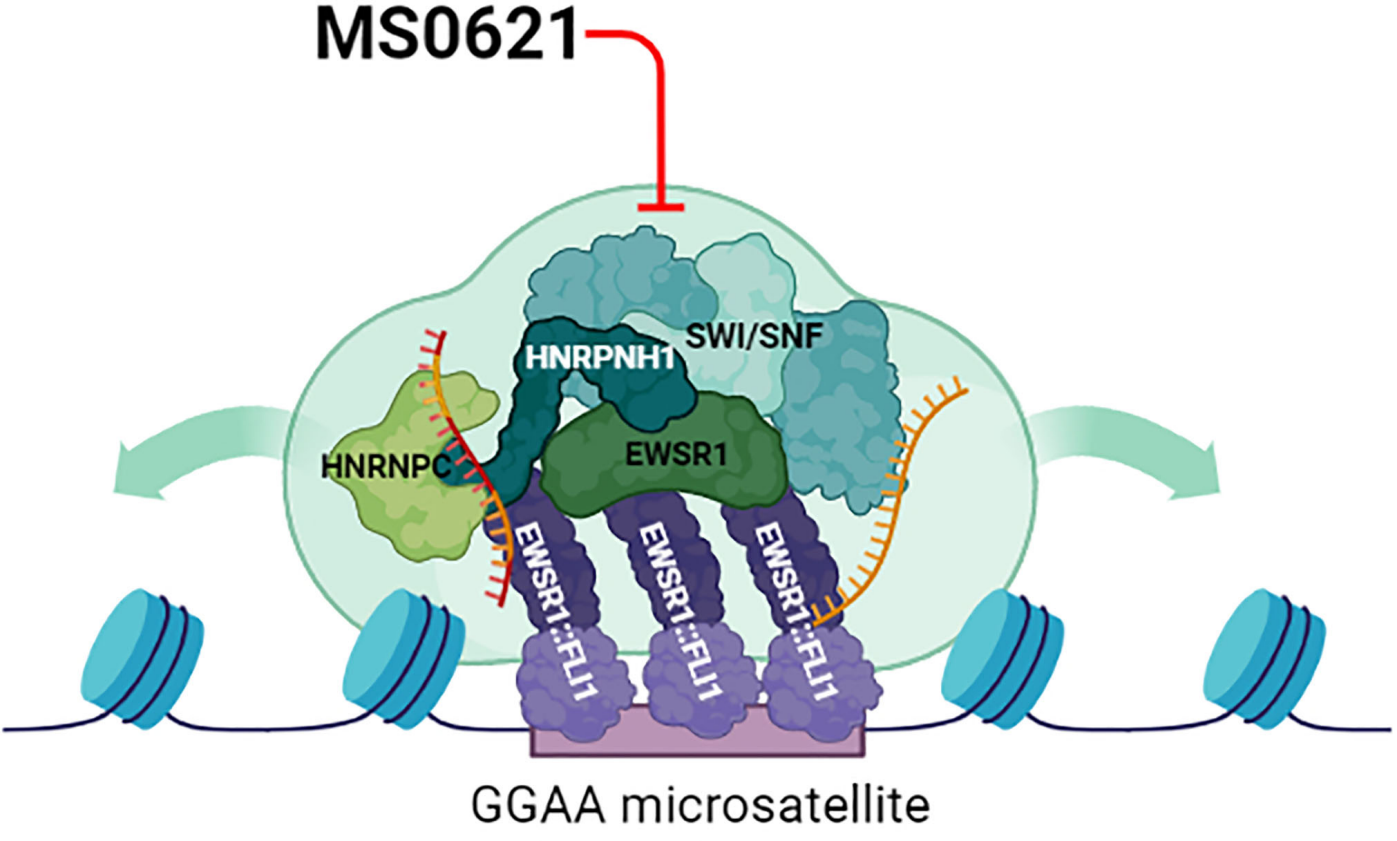
Proposed Model of Perturbation of MS0621. The chromatin landscape at EWSR1::FLI1 targeted sites is likely established and maintained through the activity of SWI/SNF as well as through biophysical properties such as phase separation mediated by proteins and RNA (gold RNA molecule). Likewise, EWSR1::FLI1 alters the function of RNA binding proteins to program Ewing sarcoma-specific alternative patterns (gold and red RNA molecule). EWSR1::FLI1 may depend on the complex to mediate its roles in chromatin and transcription, but this dependence may also represent a vulnerability for Ewing sarcoma cells. MS0621 interacts with and may perturb the complex and inhibit the functions required by EWSR1::FLI1 leading to altered chromatin organization, gene expression, and splicing programs. Thus, perturbation of these functions likely contributes to the cell proliferation defects and cell cycle arrest observed in Ewing sarcoma cell lines upon MS0621 treatment. Created with BioRender.com.

MS0621 interacted with a complex that included EWSR1::FLI1 and multiple chromatin-binding and RNA-associated proteins. The inclusion of EWSR1::FLI1 in an MS0621-interacting complex may indicate that the fusion oncoprotein interacts with the complex to co-opt its normal function (**Figure 6**). For example, EWSR1::FLI1 inhibits RNA splicing mediated by interactions of EWSR1 and YBX1, SRSF proteins, and hnRNPA1 and antagonizes a subset of splicing events co-regulated by FLI1 and the splicing regulator RBFOX2 (38, 52–55). EWSR1::FLI1, together with these proteins and several others we identify as interacting with MS0621 participate in phase separation, which may mediate its oncogenic functions. A dependency on this complex may mediate the susceptibility of Ewing sarcoma cells to MS0621 (59–62). The N-terminal low complexity domains of EWSR1::FLI1 are thought to mediate critical functions of EWSR1::FLI1 including recruitment of the SWI/SNF complex to GGAA microsatellite and the formation of phase separated condensates implicated in enhancing protein-protein interactions and regulating the expression of EWSR1::FLI1 target genes (7, 63–65). Perturbing these EWSR1::FLI1-containing condensates through overexpression of the low complexity domains of EWSR1 or TAF15 decreases the expression of EWSR1::FLI1-regulated genes without displacing EWSR1::FLI1 from chromatin (66, 67). Additionally, altering the ability of EWSR1::FLI1 to participate in phase separation by mutating the critical tyrosine residues in the N-terminus decreases SWI/SNF recruitment, chromatin accessibility, and deposition of enhancer-associated histone PTMs at EWSR1::FLI1-bound GGAA loci, and inhibits the expression of EWSR1::FLI1-regulated genes (7). Similarly, mutating tyrosine residues in one of hnRNPH1’s low complexity domains alters its phase separation properties and the splicing of hnRNPH1-regulated exons (68). As hnRNPH1 is implicated in the splicing of EWSR1 and altering of EWSR1 levels can affect EWSR1::FLI1 function, perturbation or loss of hnRNPH1 may modulate EWSR1::FLI1-mediated phenotypes through multiple mechanisms (69). Thus, EWSR1::FLI1 recruits and may be dependent on the complex, but perturbation the complex or its members may also be a sensitivity of Ewing sarcoma (**Figure 6**). The dependency on a complex involving EWSR1::FLI1, in particular in the context of a specific chromatin or developmental state, may contribute to the differential sensitivity of Ewing sarcoma cell lines to MS0621 compared to cells of other lineages.

MS0621 decreases FAIRE signal at EWSR1::FLI1-bound loci without displacing the oncoprotein from chromatin, indicating that EWSR1::FLI1 may be necessary but not sufficient for maintaining FAIRE signal at its target loci. Although we conceived of our screen as a strategy to explore chromatin accessibility, we have also demonstrated that FAIRE signal can be detected at chromatin regions without displaced nucleosomes, possibly due to unstable nucleosomes (70). It is possible that MS0621 modulates an activity that controls chromatin states without affecting nucleosome displacement. Together, these data support a broader influence on transcription and chromatin states for MS0621 than EWSR1::FLI1 and point to a role for MS0621 in modulating transcription-linked processes that affect chromatin states.

Despite our efforts to identify a single direct target of MS0621, we were unable to further dissect the complexes using biochemical techniques. Among chromatin-associated proteins, MS0621 interacted with members of the SWI/SNF chromatin remodeling complex, which has recently been implicated in EWSR1::FLI1-mediated chromatin remodeling (7). ARID1A and ARID1B are mutually exclusive SWI/SNF subunits, with ARID1B containing complexes associated with repressed chromatin (25, 26). In contrast to EWSR1::FLI1, which interacts only with ARID1A-containing SWI/SNF complexes, MS0621 interacts with both ARID1A- and ARID1B-containing SWI/SNF complexes. These interactions suggest that MS0621 associates with multiple complexes, one of which includes EWSR1::FLI1. It is unlikely that EWSR1::FLI1 mediates the interaction of MS0621 with these proteins, as interactions with similar proteins were identified in cells that do not express the fusion oncoprotein. Interestingly, we noted that EWSR1::ATF1 oncoprotein interacted with MS0621, whereas native ATF1 did not in non-clear cell sarcoma cells. These data suggest that the EWSR1 domain mediates the interactions of the EWSR1-fusion oncoproteins with the MS0621-targeted complex.

MS0621-interacting complexes include RNA, and the inclusion of some constituents of the MS0621-interacting complex were mediated by RNA. However, interactions with EWSR1::FLI1, hnRNPH1, and EWSR1 were independent of RNA and resistant to high salt. This tight interaction supports that these proteins constitute core members of the complex. In contrast, the interactions between DHX9 and YBX1, proteins with known roles in Ewing sarcoma, were greatly attenuated in the presence of high salt and RNase A (55, 71, 72). These data suggest that neither DHX9 nor YBX1 are core members of the MS0621-interacting complex and indicate that the effects of MS0621 are unlikely to mediated through these proteins. Modest knockdown of hnRNPH1 phenocopied the effect of MS0621 on cell proliferation and decreased FAIRE signal to a similar magnitude but at a greater set of loci. These data are consistent with the recent identification of hnRNPH2, a closely related paralog of hnRNPH1, as one of six splicing factors with roles in splice selection in Ewing sarcoma (56). Interestingly, MS0621 and hnRNPH1 knockdown decreased FAIRE signal at EWSR1::FLI1-bound loci that were not microsatellites. These data support a link between hnRNPs and chromatin state as measured by FAIRE. As hnRNPH1 knockdown altered FAIRE signal at more regions than MS0621, these data also suggest that perturbation of an individual RNA binding protein in the MS0621-interacting complex broadly affects the chromatin states accessibility landscape. Together, these data suggest that MS0621 perturbs processes involving activities of EWSR1::FLI1 on chromatin states, transcription, and splicing.

The effects of EWSR1::FLI1 on chromatin states, transcription, and alternative splicing have been long appreciated (5–8, 24, 38, 52–57). MS0621 reprograms the EWSR1::FLI1-regulated alternative splicing pattern, decreasing tumor-characteristic intron retention and altering the inclusion of exons harboring highly conserved sequences. Indeed, SE events associated with the loss of EWSR1::FLI1 were significantly enriched among those gained with MS0621 treatment. Interestingly, MS0621 treatment and EWSR1::FLI1 knockdown (38, 57) altered the splicing of the closely related CLK1 and CLK4 kinases which have been implicated in the regulation of alternative splicing through phosphorylation of SRSF proteins (73–77). Of note, CLK1 also regulates the splicing of factors with roles in cell cycle progression, suggesting an additional method by which MS0621 may influence gene expression and the cell cycle (78). Similarly, MS0621 influences splicing of the histone methyltransferases EHMT1 (GLP), EZH2, NSD2, and SETD2, enzymes associated with transcription and chromatin organization. Alterations in the splicing of these genes suggests that MS0621 perturbs elements of the splicing machinery associated with EWSR1::FLI1.

By screening for compounds that modulate an oncogenic chromatin state, we identified MS0621 as a novel inhibitor of oncoprotein-driven aberrant chromatin organization and transcription in Ewing sarcoma. Toxicities of the compound in animals limited extensive preclinical evaluation. The specific mechanism underlying this effect remains a focus of ongoing investigation. Identification of a specific targets would enable necessary structure-function experiments to optimize on-target effects while minimizing toxicity. Using MS0621, we identified a previously unappreciated relationship between RNA binding proteins and RNA splicing in modulating chromatin states in Ewing sarcoma. This study illustrates the reciprocal influence of epigenetic and transcriptomic regulation in cancer and provides a framework for targeting chromatin states in future drug discovery efforts.

## Data availability statement

The datasets presented in this study can be found in online repositories. The names of the repository/repositories and accession number(s) can be found below: https://www.ncbi.nlm.nih.gov/geo/, GSE213545.

## Author contributions

TV: Data curation, formal analysis, investigation, visualization, methodology, writing. AW: Data curation, formal analysis, investigation. JF: Formal analysis, investigation, visualization. SM: Formal analysis, investigation, visualization. AWM: Data curation, investigation. VN: Data curation, investigation, visualization. BB: Data curation, investigation, visualization. KL: Reagent generation. KB: Reagent generation. JJ: Supervision, methodology. LJ: Supervision, methodology. SF: Supervision, methodology. ALM: Data curation, formal analysis, investigation. SP: Conceptualization, resources, data curation, formal analysis. ID: Conceptualization, resources, data curation, formal analysis, supervision, funding acquisition, writing–original draft, project administration, writing–review and editing. All authors contributed to the article and approved the submitted version.

## Supplementary material

Supplementary Figure 1
**(A)** MS0621 does not decrease EWSR1::FLI1 expression. EWSR1::FLI1 expression measured by qRT-PCR in EWS894 and EWS502 cells following treatment with 5 μM MS0621 or vehicle control (PBS) for 16 h. Results are shown as the fold change of vehicle treated cells. Error bars represent the standard deviation of three biological replicates. **(B)** MS0621 does not influence EWSR1::FLI1 protein levels. EWSR1::FLI1 protein abundance measured by western blot in EWS894 and EWS502 cells following treatment with 5 μM MS0621 or vehicle control (PBS) for 16 h. Top: representative western blots of EWSR1::FLI1 and α-Tubulin control. Bottom: EWSR1::FLI1 intensity was normalized to tubulin. Results are shown as the fold change of vehicle treated cells. Error bars represent the standard deviation of three biological replicates.

Supplementary Figure 2
**(A)** Cell proliferation assessed by live cell imaging for MHH-ES cells treated with MS0621 or vehicle control over 6 days. Proliferation was assessed by images captured every 2 hours. Results are shown as the percent confluence. Error bars represent the standard error of the mean for six (MS0621 treatment) technical replicates or two (vehicle control) technical replicates. **(B)** MS0621 does not induce apoptosis in Ewing sarcoma cells. Following 3 days of treatment with 10 μM MS0621, EWS894 cells were stained with Annexin V and propidium iodide (PI) and analyzed by flow cytometry. Cells treated with staurosporine for 4 hours were used as a positive control. **(C)** MS0621 inhibits Ewing sarcoma cell division. EWS502 cells were stained with CFSE dye and divided into 3 conditions: untreated, 5 μM MS0621, or vehicle (DMSO). Cells were harvested, fixed, and analyzed by flow cytometry. MS0621- and vehicle-treated cells were compared to the untreated stained control. **(D)** MS0621 increases the proportion of cells in G0/G1 and decreases the proportion of cells in S-phase. Following treatment of EWS502 cells with 5 μM MS0621 or vehicle (DMSO) for the indicated time, cells were stained with propidium iodide (PI) and analyzed by flow cytometry. Right: Quantification of the proportion of cells in each stage of the cell cycle

Supplementary Figure 3
**(A)** Log2 RCI for MS0621 (blue), MS0621 analogs (gray), putative G9a inhibitors (black), DMSO control and UNC0638 (far right) for screening plate 1. **(B)** Cell proliferation curves for EWS894 cells treated with the indicated compounds (threefold dilutions from 10 μM) or vehicle control for 3 days. Proliferation was assessed on day 3 by Cell Titer Glo assay. Results are shown as the percent of vehicle treated cells. Error bars represent the SD of three technical replicates. **(C)** Di-methylation of histone H3 lysine 9 measured by western blot in EWS894 cells following treatment with the indicated concentration of MS0621, UNC0638, or vehicle control (DMSO) for 16 hours. Top: representative western blots of H3K9me2 and histone H3 loading control. Bottom: Quantification of western blot band intensities for H3K9me2 normalized to histone H3 band intensities. Results are shown as the fold change of vehicle treated cells. Error bars represent the standard deviation of two biological replicates. **(D)** Cell proliferation curves for EWS894 cells treated with the indicated compounds (twofold dilutions from 5 μM) or vehicle control for 3 days. Proliferation was assessed on day 3 by Cell Titer Glo assay. Results are shown as the percent of vehicle treated cells. Error bars represent the SD of three technical replicates. **(E)** Cell proliferation curves for EWS894 cells treated with the indicated doses of MS0621 or UNC4151 (twofold dilutions from 5 μM to 0.15625 μM) or vehicle control for 3 days. Proliferation was assessed on day 3 by WST assay. Results are shown as the fold change of vehicle treated cells. Error bars represent the SD of three biological replicates. **(F)** STRING diagram displaying interactions networks between proteins identified by mass spectrometry in HEK293T cells. **(G)** KEGG Enriched Biological Processes GO terms for proteins identified by mass spectrometry in HEK293T cells. Results shown are the -log_10_ FDR for the top 10% of enriched GO terms. **(H)** Western blot analyses of proteins enriched by 5 μM MS1360 (+) or vehicle control (-) in MNase-digested nuclear extracts of HEK293T cells with or without 100 ug/mL RNase A digestion assessed by western blot. 20% of the MNase-digested nuclear extract input was included for reference. **(I)** Proteins enriched by 5 μM MS1360 (+) or vehicle control (-) in the indicated extracts of A673 cells with or without digestion with 10 U RNase H assessed by western blot. 20% of the extract input was included for reference. **(J)** Western blot analyses of proteins enriched 5 μM MS1360 (+) or vehicle control (-) in MNase-digested nuclear extracts of A673, EWS502, SU-CCS1, and 786-O cells assessed by western blot. 20% of the MNase-digested nuclear extract input was included for reference. **(K)** Western blot analyses of proteins co-immunoprecipitated by EWSR1::FLI1 in MNase-digested nuclear extracts of A673 cells. **(L)** Representative western blot analyses for protein enrichment under standard (150 mM NaCl) and high salt (500 mM NaCl) conditions normalized to Input band intensities quantified in . Results shown are representative of three technical replicates.

Supplementary Figure 4
**(A)** Western blot analyses of EWSR1::FLI1 and hnRNPH1 protein following infection with the indicated sh RNAs in A673-CRISPRi cells for 4 days. Left: Representative western blots. Right: Quantification of western blot band intensities for EWSR1::FLI1 and hnRNPH1 normalized to Histone H3 band intensities. Results are shown as the fold change of non-specific guide (sh NS) cells. Error bars represent the standard deviation of three biological replicates. **(B)** EWSR1::FLI1 and hnRNPH1 expression measured by RT-qPCR in A673-CRISPRi cells following infection with the indicated sh RNAs for 4 days. Results are shown as the fold change of non-specific guide (sh NS) cells. Error bars represent the standard deviation of three biological replicates. **(C)** FAIRE-qPCR at EWSR1::FLI1-bound and control loci in A673-CRISPRi cells infected with the indicated sg and sh RNAs for 4 days. Results are shown as a fraction of input control. Error bars represent the standard deviation of three biological replicates. **(D)** Cell proliferation curves by live cell imaging for EWS502-CRISPRi cells infected with the indicated sgRNAs for 4 days. Proliferation was assessed by images captured every 2 hours. Results are shown as the percent confluence. Error bars represent the standard error of the mean of 25 images taken from one well per condition per time point. **(E)** FAIRE-qPCR at EWSR1::FLI1-bound loci in EWS502-CRISPRi cells infected with the indicated sgRNAs (Top: sg hnRNPH1, Bottom: sg EWSR1) for 4 days. Results are shown as a fraction of input control. Error bars represent the standard error of two biological replicates. **(F)** FAIRE-qPCR at positive control loci in A673-CRISPRi cells infected with the indicated sgRNAs (Top: sg hnRNPH1, Bottom: sg EWSR1) for 4 days. Results are shown as a fraction of input control. Error bars represent the standard error of three biological replicates.

Supplementary Figure 5
**(A)** Volcano plot of log2 fold change of gene expression in EWS894 cells treated 16 hours with 5 μM MS0621 or vehicle control (DMSO). Colored points represent genes that are both upregulated by EWSR1::FLI1 per Kinsey et al., 2006 and differentially expressed with MS0621 treatment. **(B)** Enriched Biological Processes GO terms for genes that are both downregulated with MS0621 treatment and upregulated by EWSR1::FLI1. Results shown are the -log_10_ adjusted p-value for the top 20 enriched GO terms. **(C)** Enriched Biological Processes GO terms for all genes downregulated with MS0621 treatment. Results shown are the -log_10_ adjusted p-value for the top 20 enriched GO terms. **(D)** Enriched Biological Processes GO terms for all genes upregulated with MS0621 treatment. Results shown are the -log_10_ adjusted p-value for the top 20 enriched GO terms. **(E)** Enriched Biological Processes GO terms for genes with RI events lost with MS0621 treatment. Results shown are the -log_10_ adjusted p-value for enriched GO terms. **(F)** Enriched Biological Processes GO terms for genes with SE events in NMD-associated transcripts events lost with MS0621 treatment. Results shown are the -log_10_ adjusted p-value for enriched GO terms. **(G)** Significant differential alternative splicing identified by rMATS in A673 following EWSR1::FLI1 or control knockdown for 48 hr and meeting the additional criteria: supported by at least 20 reads, Inclusion Level Difference > 10%, and FDR < 0.05. Analyzed data were previously published (8).

## References

[1] Plass C Pfister SM Lindroth AM Bogatyrova O Claus R Lichter P. Mutations in regulators of the epigenome and their connections to global chromatin patterns in cancer. Nat Rev Genet (2013) 14(11):765–80. doi: 10.1038/nrg3554 24105274

[2] Morgan MA Shilatifard A. Chromatin signatures of cancer. Genes Dev (2015) 29(3):238–49. doi: 10.1101/gad.255182.114 PMC4318141 25644600

[3] Delattre O Zucman J Melot T Garau XS Zucker JM Lenoir GM. The Ewing family of tumors–a subgroup of small-round-cell tumors defined by specific chimeric transcripts. N Engl J Med (1994) 331(5):294–9. doi: 10.1056/NEJM199408043310503 8022439

[4] Grünewald TGP Cidre-Aranaz F Surdez D Tomazou EM de Álava E Kovar H. Ewing Sarcoma. Nat Rev Dis Primers. (2018) 4(1):5. doi: 10.1038/s41572-018-0003-x 29977059

[5] Gangwal K Sankar S Hollenhorst PC Kinsey M Haroldsen SC Shah AA. Microsatellites as EWS/FLI response elements in ewing’s sarcoma. Proc Natl Acad Sci U S A. (2008) 105(29):10149–54. doi: 10.1073/pnas.0801073105 PMC2481306 18626011

[6] Patel M Simon JM Iglesia MD Wu SB McFadden AW Lieb JD. Tumor-specific retargeting of an oncogenic transcription factor chimera results in dysregulation of chromatin and transcription. Genome Res (2012) 22(2):259–70. doi: 10.1101/gr.125666.111 PMC3266033 22086061

[7] Boulay G Sandoval GJ Riggi N Iyer S Buisson R Naigles B. Cancer-specific retargeting of BAF complexes by a prion-like domain. Cell (2017) 171(1):163–78.e19. doi: 10.1016/j.cell.2017.07.036 28844694 PMC6791823

[8] Riggi N Knoechel B Gillespie SM Rheinbay E Boulay G Suvà ML. EWS-FLI1 utilizes divergent chromatin remodeling mechanisms to directly activate or repress enhancer elements in Ewing sarcoma. Cancer Cell (2014) 26(5):668–81. doi: 10.1016/j.ccell.2014.10.004 PMC4492343 25453903

[9] Pattenden SG Simon JM Wali A Jayakody CN Troutman J McFadden AW. High-throughput small molecule screen identifies inhibitors of aberrant chromatin accessibility. Proc Natl Acad Sci U S A. (2016) 113(11):3018–23. doi: 10.1073/pnas.1521827113 PMC4801272 26929321

[10] Slaughter MJ Shanle EK McFadden AW Hollis ES Suttle LE Strahl BD. PBRM1 bromodomains variably influence nucleosome interactions and cellular function. J Biol Chem (2018) 293(35):13592–603. doi: 10.1074/jbc.RA118.003381 PMC6120218 29986887

[11] Gilbert LA Horlbeck MA Adamson B Villalta JE Chen Y Whitehead EH. Genome-scale CRISPR-mediated control of gene repression and activation. Cell (2014) 159(3):647–61. doi: 10.1016/j.cell.2014.09.029 PMC4253859 25307932

[12] Simon JM Giresi PG Davis IJ Lieb JD. Using formaldehyde-assisted isolation of regulatory elements (FAIRE) to isolate active regulatory DNA. Nat Protoc (2012) 7(2):256–67. doi: 10.1038/nprot.2011.444 PMC3784247 22262007

[13] Livak KJ Schmittgen TD. Analysis of relative gene expression data using real-time quantitative PCR and the 2(-delta delta C(T)) method. Methods (2001) 25(4):402–8. doi: 10.1006/meth.2001.1262 11846609

[14] Ruthenburg AJ Li H Milne TA Dewell S McGinty RK Yuen M. Recognition of a mononucleosomal histone modification pattern by BPTF *via* multivalent interactions. Cell (2011) 145(5):692–706. doi: 10.1016/j.cell.2011.03.053 21596426 PMC3135172

[15] Antrobus R Borner GH. Improved elution conditions for native co-immunoprecipitation. PloS One (2011) 6(3):e18218. doi: 10.1371/journal.pone.0018218 21448433 PMC3063181

[16] Love MI Huber W Anders S. Moderated estimation of fold change and dispersion for RNA-seq data with DESeq2. Genome Biol (2014) 15(12):550. doi: 10.1186/s13059-014-0550-8 25516281 PMC4302049

[17] Wu T Hu E Xu S Chen M Guo P Dai Z. clusterProfiler 4.0: A universal enrichment tool for interpreting omics data. Innovation (Camb) (2021) 2(3):100141. doi: 10.1016/j.xinn.2021.100141 34557778 PMC8454663

[18] Shen S Park JW Lu ZX Lin L Henry MD Wu YN. rMATS: robust and flexible detection of differential alternative splicing from replicate RNA-seq data. Proc Natl Acad Sci U S A. (2014) 111(51):E5593–601. doi: 10.1073/pnas.1419161111 PMC4280593 25480548

[19] Lee S Cook D Lawrence M. Plyranges: a grammar of genomic data transformation. Genome Biol (2019) 20(1):4. doi: 10.1186/s13059-018-1597-8 30609939 PMC6320618

[20] Möller E Praz V Rajendran S Dong R Cauderay A Xing YH. EWSR1-ATF1 dependent 3D connectivity regulates oncogenic and differentiation programs in clear cell sarcoma. Nat Commun (2022) 13(1):2267. doi: 10.1038/s41467-022-29910-4 35477713 PMC9046276

[21] Chakraborty P Grosse F. Human DHX9 helicase preferentially unwinds RNA-containing displacement loops (R-loops) and G-quadruplexes. DNA Repair (Amst). (2011) 10(6):654–65. doi: 10.1016/j.dnarep.2011.04.013 21561811

[22] Cristini A Groh M Kristiansen MS Gromak N. RNA/DNA hybrid interactome identifies DXH9 as a molecular player in transcriptional termination and r-Loop-Associated DNA damage. Cell Rep (2018) 23(6):1891–905. doi: 10.1016/j.celrep.2018.04.025 PMC5976580 29742442

[23] Wang IX Grunseich C Fox J Burdick J Zhu Z Ravazian N. Human proteins that interact with RNA/DNA hybrids. Genome Res (2018) 28(9):1405–14. doi: 10.1101/gr.237362.118 PMC6120628 30108179

[24] Selvanathan SP Graham GT Grego AR Baker TM Hogg JR Simpson M. EWS-FLI1 modulated alternative splicing of ARID1A reveals novel oncogenic function through the BAF complex. Nucleic Acids Res (2019) 47(18):9619–36. doi: 10.1093/nar/gkz699 PMC6765149 31392992

[25] Raab JR Resnick S Magnuson T. Genome-wide transcriptional regulation mediated by biochemically distinct SWI/SNF complexes. PloS Genet (2015) 11(12):e1005748. doi: 10.1371/journal.pgen.1005748 26716708 PMC4699898

[26] Pagliaroli L Porazzi P Curtis AT Scopa C Mikkers HMM Freund C. Inability to switch from ARID1A-BAF to ARID1B-BAF impairs exit from pluripotency and commitment towards neural crest formation in ARID1B-related neurodevelopmental disorders. Nat Commun (2021) 12(1):6469. doi: 10.1038/s41467-021-26810-x 34753942 PMC8578637

[27] Böhm V Hieb AR Andrews AJ Gansen A Rocker A Tóth K. Nucleosome accessibility governed by the dimer/tetramer interface. Nucleic Acids Res (2011) 39(8):3093–102. doi: 10.1093/nar/gkq1279 PMC3082900 21177647

[28] Stacks PC Schumaker VN. Nucleosome dissociation and transfer in concentrated salt solutions. Nucleic Acids Res (1979) 7(8):2457–67. doi: 10.1093/nar/7.8.2457 PMC342396 523323

[29] Henikoff S Henikoff JG Sakai A Loeb GB Ahmad K. Genome-wide profiling of salt fractions maps physical properties of chromatin. Genome Res (2009) 19(3):460–9. doi: 10.1101/gr.087619.108 PMC2661814 19088306

[30] Kinsey M Smith R Lessnick SL. NR0B1 is required for the oncogenic phenotype mediated by EWS/FLI in ewing’s sarcoma. Mol Cancer Res (2006) 4(11):851–9. doi: 10.1158/1541-7786.MCR-06-0090 17114343

[31] García-Aragoncillo E Carrillo J Lalli E Agra N Gómez-López G Pestaña A. DAX1, a direct target of EWS/FLI1 oncoprotein, is a principal regulator of cell-cycle progression in ewing’s tumor cells. Oncogene (2008) 27(46):6034–43. doi: 10.1038/onc.2008.203 18591936

[32] Miyagawa Y Okita H Nakaijima H Horiuchi Y Sato B Taguchi T. Inducible expression of chimeric EWS/ETS proteins confers ewing’s family tumor-like phenotypes to human mesenchymal progenitor cells. Mol Cell Biol (2008) 28(7):2125–37. doi: 10.1128/MCB.00740-07 PMC2268432 18212050

[33] Baliko F Bright T Poon R Cohen B Egan SE Alman BA. Inhibition of notch signaling induces neural differentiation in Ewing sarcoma. Am J Pathol (2007) 170(5):1686–94. doi: 10.2353/ajpath.2007.060971 PMC1854963 17456774

[34] Jayabal P Zhou F Lei X Ma X Blackman B Weintraub ST. NELL2-cdc42 signaling regulates BAF complexes and Ewing sarcoma cell growth. Cell Rep (2021) 36(1):109254. doi: 10.1016/j.celrep.2021.109254 34233189 PMC8312579

[35] Rota R Ciarapica R Miele L Locatelli F. Notch signaling in pediatric soft tissue sarcomas. BMC Med (2012) 10:141. doi: 10.1186/1741-7015-10-141 23158439 PMC3520771

[36] Musa J Cidre-Aranaz F Aynaud MM Orth MF Knott MML Mirabeau O. Cooperation of cancer drivers with regulatory germline variants shapes clinical outcomes. Nat Commun (2019) 10(1):4128. doi: 10.1038/s41467-019-12071-2 31511524 PMC6739408

[37] Ohmura S Marchetto A Orth MF Li J Jabar S Ranft A. Translational evidence for RRM2 as a prognostic biomarker and therapeutic target in Ewing sarcoma. Mol Cancer. (2021) 20(1):97. doi: 10.1186/s12943-021-01393-9 34315482 PMC8314608

[38] Saulnier O Guedri-Idjouadiene K Aynaud MM Chakraborty A Bruyr J Pineau J. ERG transcription factors have a splicing regulatory function involving RBFOX2 that is altered in the EWS-FLI1 oncogenic fusion. Nucleic Acids Res (2021) 49(9):5038–56. doi: 10.1093/nar/gkab305 PMC8136815 34009296

[39] Dvinge H Bradley RK. Widespread intron retention diversifies most cancer transcriptomes. Genome Med (2015) 7(1):45. doi: 10.1186/s13073-015-0168-9 26113877 PMC4480902

[40] Jung H Lee D Lee J Park D Kim YJ Park WY. Intron retention is a widespread mechanism of tumor-suppressor inactivation. Nat Genet (2015) 47(11):1242–8. doi: 10.1038/ng.3414 26437032

[41] Braunschweig U Barbosa-Morais NL Pan Q Nachman EN Alipanahi B Gonatopoulos-Pournatzis T. Widespread intron retention in mammals functionally tunes transcriptomes. Genome Res (2014) 24(11):1774–86. doi: 10.1101/gr.177790.114 PMC4216919 25258385

[42] Ni JZ Grate L Donohue JP Preston C Nobida N O’Brien G. Ultraconserved elements are associated with homeostatic control of splicing regulators by alternative splicing and nonsense-mediated decay. Genes Dev (2007) 21(6):708–18. doi: 10.1101/gad.1525507 PMC1820944 17369403

[43] Nickless A Bailis JM You Z. Control of gene expression through the nonsense-mediated RNA decay pathway. Cell Biosci (2017) 7:26. doi: 10.1186/s13578-017-0153-7 28533900 PMC5437625

[44] Lareau LF Inada M Green RE Wengrod JC Brenner SE. Unproductive splicing of SR genes associated with highly conserved and ultraconserved DNA elements. Nature (2007) 446(7138):926–9. doi: 10.1038/nature05676 17361132

[45] Park SK Zhou X Pendleton KE Hunter OV Kohler JJ O’Donnell KA. A conserved splicing silencer dynamically regulates O-GlcNAc transferase intron retention and O-GlcNAc homeostasis. Cell Rep (2017) 20(5):1088–99. doi: 10.1016/j.celrep.2017.07.017 PMC5588854 28768194

[46] Ge Y Porse BT. The functional consequences of intron retention: alternative splicing coupled to NMD as a regulator of gene expression. Bioessays (2014) 36(3):236–43. doi: 10.1002/bies.201300156 24352796

[47] Bejerano G Pheasant M Makunin I Stephen S Kent WJ Mattick JS. Ultraconserved elements in the human genome. Science (2004) 304(5675):1321–5. doi: 10.1126/science.1098119 15131266

[48] French CE Wei G Lloyd JPB Hu Z Brooks AN Brenner SE. Transcriptome analysis of alternative splicing-coupled nonsense-mediated mRNA decay in human cells reveals broad regulatory potential. bioRxiv (2020). doi: 10.1101/2020.07.01.183327

[49] Leclair NK Brugiolo M Urbanski L Lawson SC Thakar K Yurieva M. Poison exon splicing regulates a coordinated network of SR protein expression during differentiation and tumorigenesis. Mol Cell (2020) 80(4):648–65.e9. doi: 10.1016/j.molcel.2020.10.019 33176162 PMC7680420

[50] Titus MB Chang AW Olesnicky EC. Exploring the diverse functional and regulatory consequences of alternative splicing in development and disease. Front Genet (2021) 12:775395. doi: 10.3389/fgene.2021.775395 34899861 PMC8652244

[51] Tan ZW Fei G Paulo JA Bellaousov S Martin SES Duveau DY. O-GlcNAc regulates gene expression by controlling detained intron splicing. Nucleic Acids Res (2020) 48(10):5656–69. doi: 10.1093/nar/gkaa263 PMC7261177 32329777

[52] Knoop LL Baker SJ. EWS/FLI alters 5’-splice site selection. J Biol Chem (2001) 276(25):22317–22. doi: 10.1074/jbc.M008950200 11301318

[53] Knoop LL Baker SJ. The splicing factor U1C represses EWS/FLI-mediated transactivation. J Biol Chem (2000) 275(32):24865–71. doi: 10.1074/jbc.M001661200 10827180

[54] Yang L Chansky HA Hickstein DD. EWS.Fli-1 fusion protein interacts with hyperphosphorylated RNA polymerase II and interferes with serine-arginine protein-mediated RNA splicing. J Biol Chem (2000) 275(48):37612–8. doi: 10.1074/jbc.M005739200 10982800

[55] Chansky HA Hu M Hickstein DD Yang L. Oncogenic TLS/ERG and EWS/Fli-1 fusion proteins inhibit RNA splicing mediated by YB-1 protein. Cancer Res (2001) 61(9):3586–90. 11325824

[56] Graham GT Selvanathan SP Zöllner SK Stahl E Shlien A Caplen NJ. Comprehensive profiling of mRNA splicing indicates that GC content signals altered cassette exon inclusion in Ewing sarcoma. NAR Cancer (2022) 4(1):zcab052. doi: 10.1093/narcan/zcab052 35047826 PMC8759570

[57] Selvanathan SP Graham GT Erkizan HV Dirksen U Natarajan TG Dakic A. Oncogenic fusion protein EWS-FLI1 is a network hub that regulates alternative splicing. Proc Natl Acad Sci U S A. (2015) 112(11):E1307–16. doi: 10.1073/pnas.1500536112 PMC4371969 25737553

[58] Vedadi M Barsyte-Lovejoy D Liu F Rival-Gervier S Allali-Hassani A Labrie V. A chemical probe selectively inhibits G9a and GLP methyltransferase activity in cells. Nat Chem Biol (2011) 7(8):566–74. doi: 10.1038/nchembio.599 PMC3184254 21743462

[59] Ying Y Wang XJ Vuong CK Lin CH Damianov A Black DL. Splicing activation by rbfox requires self-aggregation through its tyrosine-rich domain. Cell (2017) 170(2):312–23.e10. doi: 10.1016/j.cell.2017.06.022 28708999 PMC5553710

[60] Somasekharan SP El-Naggar A Leprivier G Cheng H Hajee S Grunewald TG. YB-1 regulates stress granule formation and tumor progression by translationally activating G3BP1. J Cell Biol (2015) 208(7):913–29. doi: 10.1083/jcb.201411047 PMC4384734 25800057

[61] Lyons SM Achorn C Kedersha NL Anderson PJ Ivanov P. YB-1 regulates tiRNA-induced stress granule formation but not translational repression. Nucleic Acids Res (2016) 44(14):6949–60. doi: 10.1093/nar/gkw418 PMC5001593 27174937

[62] Liu XM Ma L Schekman R. Selective sorting of microRNAs into exosomes by phase-separated YBX1 condensates. Elife (2021) 10:e71982. doi: 10.7554/eLife.71982 PMC8612733 34766549

[63] Chong S Dugast-Darzacq C Liu Z Dong P Dailey GM Cattoglio C. Imaging dynamic and selective low-complexity domain interactions that control gene transcription. Science (2018) 361(6400). doi: 10.1126/science.aar2555 PMC6961784 29930090

[64] Johnson KM Mahler NR Saund RS Theisen ER Taslim C Callender NW. Role for the EWS domain of EWS/FLI in binding GGAA-microsatellites required for Ewing sarcoma anchorage independent growth. Proc Natl Acad Sci U S A. (2017) 114(37):9870–5. doi: 10.1073/pnas.1701872114 PMC5603999 28847958

[65] Zuo L Zhang G Massett M Cheng J Guo Z Wang L. Loci-specific phase separation of FET fusion oncoproteins promotes gene transcription. Nat Commun (2021) 12(1):1491. doi: 10.1038/s41467-021-21690-7 33674598 PMC7935978

[66] Selig EE Romero-Moreno AK Akula S Xu X Libich DS. The oncogenic fusion protein EWS-FLI1 promotes premature ageing of biomolecular condensates by catalyzing fibril formation. bioRxiv (2022). doi: 10.1101/2022.06.04.494830

[67] Chong S Graham TGW Dugast-Darzacq C Dailey GM Darzacq X Tjian R. Tuning levels of low-complexity domain interactions to modulate endogenous oncogenic transcription. Mol Cell (2022) 82(11):2084–97.e5. doi: 10.1016/j.molcel.2022.04.007 35483357

[68] Kim GH Kwon I. Distinct roles of hnRNPH1 low-complexity domains in splicing and transcription. Proc Natl Acad Sci U S A. (2021) 118(50):e2109668118. doi: 10.1073/pnas.2109668118 PMC8685725 34873036

[69] Neckles C Boer RE Aboreden N Cross AM Walker RL Kim BH. HNRNPH1-dependent splicing of a fusion oncogene reveals a targetable RNA G-quadruplex interaction. RNA (2019) 25(12):1731–50. doi: 10.1261/rna.072454.119 PMC6859848 31511320

[70] Gomez NC Hepperla AJ Dumitru R Simon JM Fang F Davis IJ. Widespread chromatin accessibility at repetitive elements links stem cells with human cancer. Cell Rep (2016) 17(6):1607–20. doi: 10.1016/j.celrep.2016.10.011 PMC5267842 27806299

[71] Dutertre M Sanchez G De Cian MC Barbier J Dardenne E Gratadou L. Cotranscriptional exon skipping in the genotoxic stress response. Nat Struct Mol Biol (2010) 17(11):1358–66. doi: 10.1038/nsmb.1912 20972445

[72] Erkizan HV Kong Y Merchant M Schlottmann S Barber-Rotenberg JS Yuan L. A small molecule blocking oncogenic protein EWS-FLI1 interaction with RNA helicase a inhibits growth of ewing’s sarcoma. Nat Med (2009) 15(7):750–6. doi: 10.1038/nm.1983 PMC2777681 19584866

[73] Martín Moyano P Němec V Paruch K. Cdc-like kinases (CLKs): Biology, chemical probes, and therapeutic potential. Int J Mol Sci (2020) 21(30):7549. doi: 10.3390/ijms21207549 PMC7593917 33066143

[74] Lindberg MF Meijer L. Dual-specificity, tyrosine phosphorylation-regulated kinases (DYRKs) and cdc2-like kinases (CLKs) in human disease, an overview. Int J Mol Sci (2021) 22(11):6047. doi: 10.3390/ijms22116047 PMC8199962 34205123

[75] Zhou Z Fu XD. Regulation of splicing by SR proteins and SR protein-specific kinases. Chromosoma (2013) 122(3):191–207. doi: 10.1007/s00412-013-0407-z 23525660 PMC3660409

[76] Duncan PI Stojdl DF Marius RM Bell JC. *In vivo* regulation of alternative pre-mRNA splicing by the Clk1 protein kinase. Mol Cell Biol (1997) 17(10):5996–6001. doi: 10.1128/MCB.17.10.5996 9315658 PMC232448

[77] Ninomiya K Kataoka N Hagiwara M. Stress-responsive maturation of Clk1/4 pre-mRNAs promotes phosphorylation of SR splicing factor. J Cell Biol (2011) 195(1):27–40. doi: 10.1083/jcb.201107093 21949414 PMC3187705

[78] Dominguez D Tsai YH Weatheritt R Wang Y Blencowe BJ Wang Z. An extensive program of periodic alternative splicing linked to cell cycle progression. Elife (2016) 5:e10288. doi: 10.7554/eLife.10288 PMC4884079 27015110

