# Supplementary figures and images for: MS0621, a novel small-molecule modulator of Ewing sarcoma chromatin accessibility, interacts with an RNA-associated macromolecular complex and influences RNA splicing
Source: Front Oncol. 2023 Jan 30;13:1099550. doi: 10.3389/fonc.2023.1099550 (PMC9924231; doi:10.3389/fonc.2023.1099550)

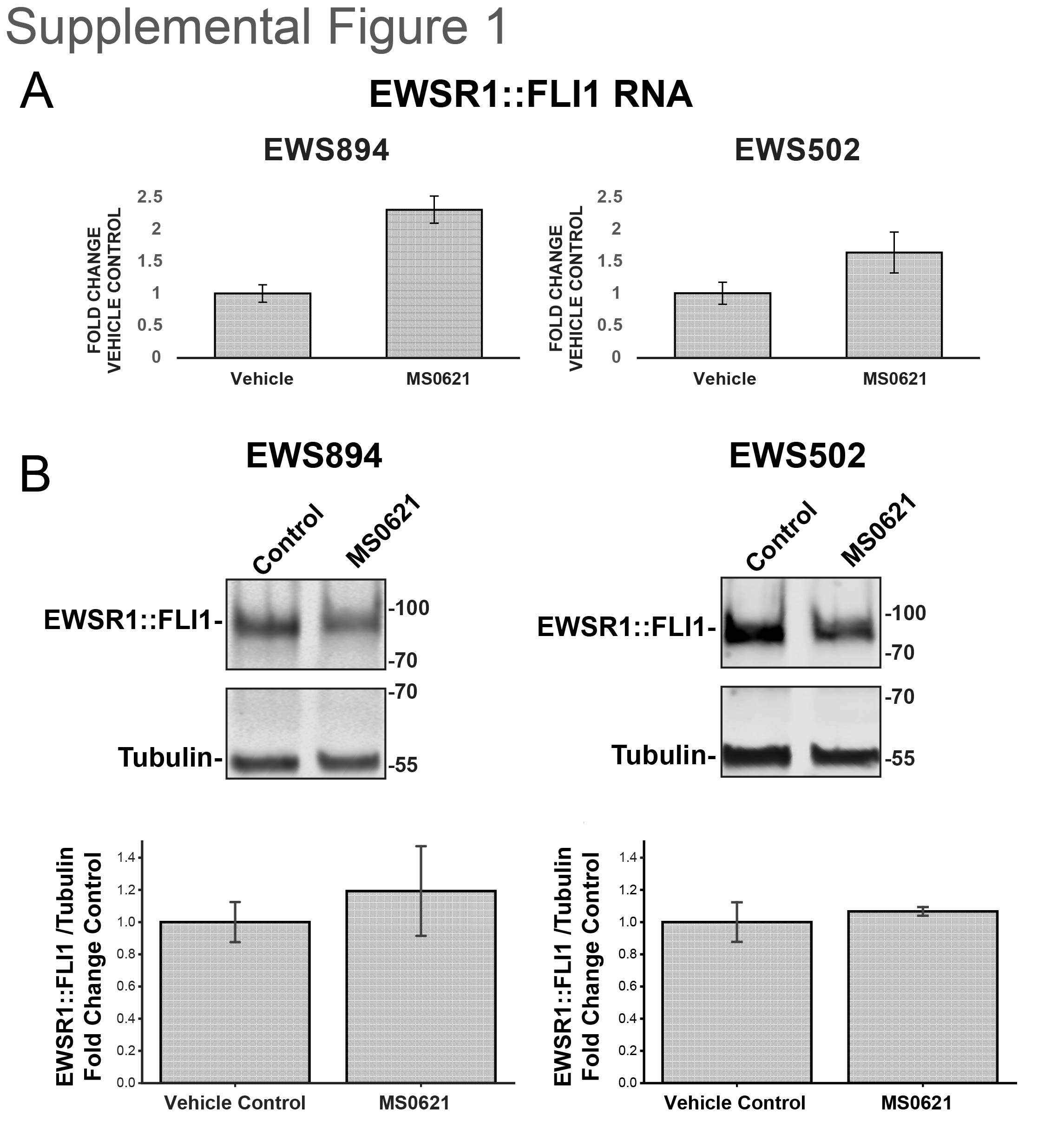

Supplement: Supplementary Figure 1 — (A) MS0621 does not decrease EWSR1::FLI1 expression. EWSR1::FLI1 expression measured by qRT-PCR in EWS894 and EWS502 cells following treatment with 5 μM MS0621 or vehicle control (PBS) for 16 h. Results are shown as the fold change of vehicle treated cells. Error bars represent the standard deviation of three biological replicates. (B) MS0621 does not influence EWSR1::FLI1 protein levels. EWSR1::FLI1 protein abundance measured by western blot in EWS894 and EWS502 cells following treatment with 5 μM MS0621 or vehicle control (PBS) for 16 h. Top: representative western blots of EWSR1::FLI1 and α-Tubulin control. Bottom: EWSR1::FLI1 intensity was normalized to tubulin. Results are shown as the fold change of vehicle treated cells. Error bars represent the standard deviation of three biological replicates. [file Image_1.jpeg]

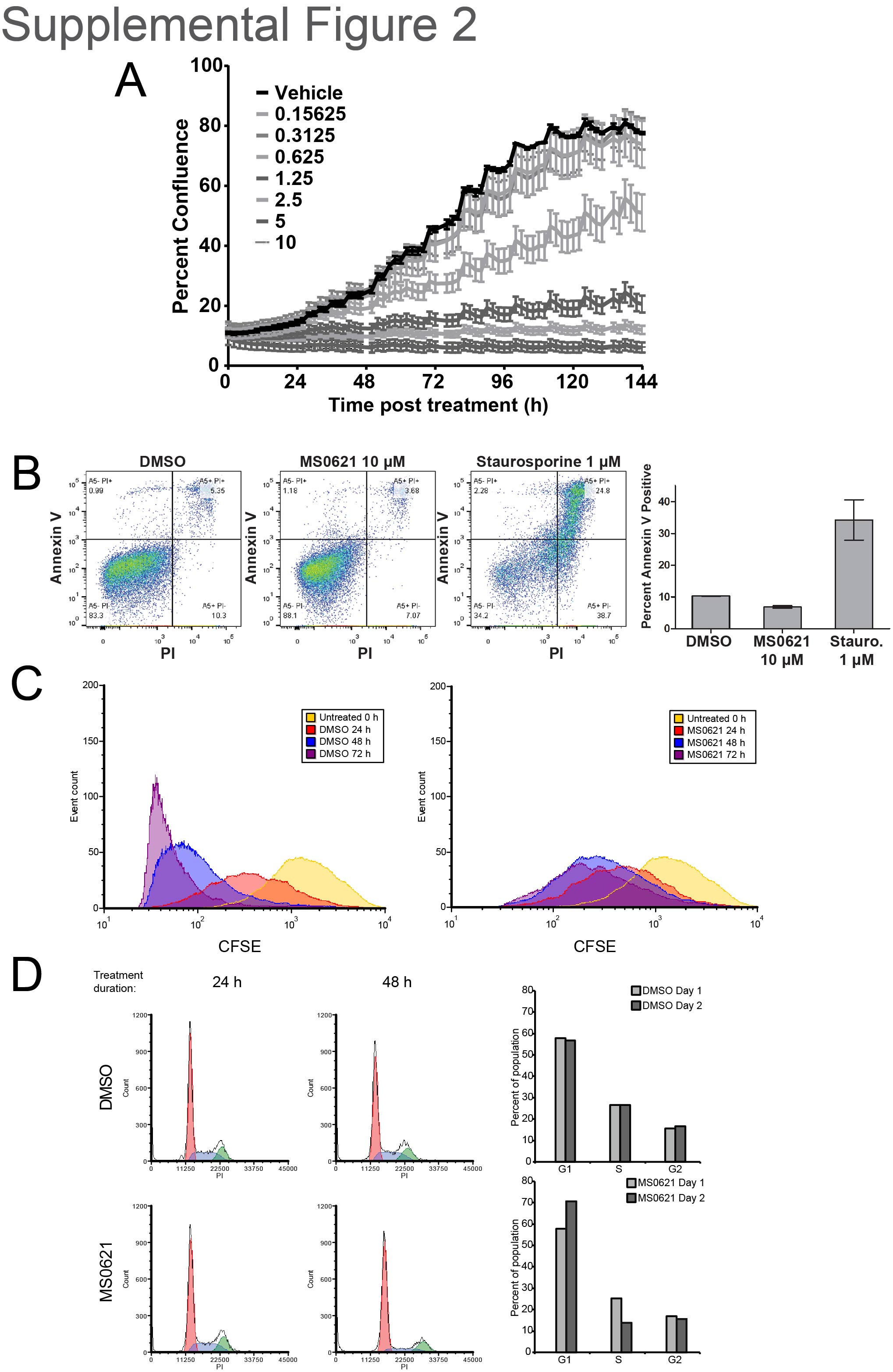

Supplement: Supplementary Figure 2 — (A) Cell proliferation assessed by live cell imaging for MHH-ES cells treated with MS0621 or vehicle control over 6 days. Proliferation was assessed by images captured every 2 hours. Results are shown as the percent confluence. Error bars represent the standard error of the mean for six (MS0621 treatment) technical replicates or two (vehicle control) technical replicates. (B) MS0621 does not induce apoptosis in Ewing sarcoma cells. Following 3 days of treatment with 10 μM MS0621, EWS894 cells were stained with Annexin V and propidium iodide (PI) and analyzed by flow cytometry. Cells treated with staurosporine for 4 hours were used as a positive control. (C) MS0621 inhibits Ewing sarcoma cell division. EWS502 cells were stained with CFSE dye and divided into 3 conditions: untreated, 5 μM MS0621, or vehicle (DMSO). Cells were harvested, fixed, and analyzed by flow cytometry. MS0621- and vehicle-treated cells were compared to the untreated stained control. (D) MS0621 increases the proportion of cells in G0/G1 and decreases the proportion of cells in S-phase. Following treatment of EWS502 cells with 5 μM MS0621 or vehicle (DMSO) for the indicated time, cells were stained with propidium iodide (PI) and analyzed by flow cytometry. Right: Quantification of the proportion of cells in each stage of the cell cycle [file Image_2.jpeg]

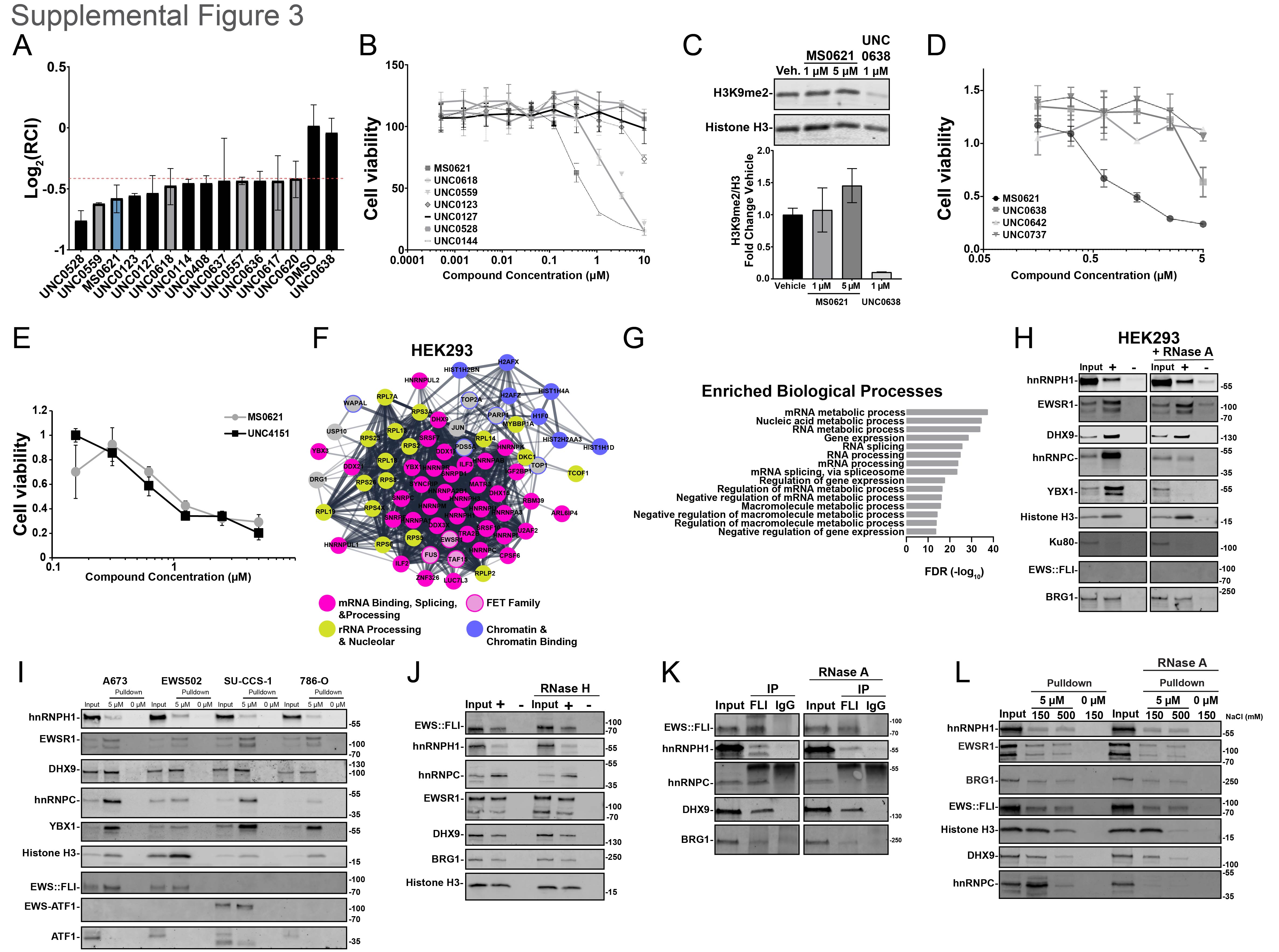

Supplement: Supplementary Figure 3 — (A) Log2 RCI for MS0621 (blue), MS0621 analogs (gray), putative G9a inhibitors (black), DMSO control and UNC0638 (far right) for screening plate 1. (B) Cell proliferation curves for EWS894 cells treated with the indicated compounds (threefold dilutions from 10 μM) or vehicle control for 3 days. Proliferation was assessed on day 3 by Cell Titer Glo assay. Results are shown as the percent of vehicle treated cells. Error bars represent the SD of three technical replicates. (C) Di-methylation of histone H3 lysine 9 measured by western blot in EWS894 cells following treatment with the indicated concentration of MS0621, UNC0638, or vehicle control (DMSO) for 16 hours. Top: representative western blots of H3K9me2 and histone H3 loading control. Bottom: Quantification of western blot band intensities for H3K9me2 normalized to histone H3 band intensities. Results are shown as the fold change of vehicle treated cells. Error bars represent the standard deviation of two biological replicates. (D) Cell proliferation curves for EWS894 cells treated with the indicated compounds (twofold dilutions from 5 μM) or vehicle control for 3 days. Proliferation was assessed on day 3 by Cell Titer Glo assay. Results are shown as the percent of vehicle treated cells. Error bars represent the SD of three technical replicates. (E) Cell proliferation curves for EWS894 cells treated with the indicated doses of MS0621 or UNC4151 (twofold dilutions from 5 μM to 0.15625 μM) or vehicle control for 3 days. Proliferation was assessed on day 3 by WST assay. Results are shown as the fold change of vehicle treated cells. Error bars represent the SD of three biological replicates. (F) STRING diagram displaying interactions networks between proteins identified by mass spectrometry in HEK293T cells. (G) KEGG Enriched Biological Processes GO terms for proteins identified by mass spectrometry in HEK293T cells. Results shown are the -log10 FDR for the top 10% of enriched GO terms. (H) Western blot analyses of p [file Image_3.jpeg]

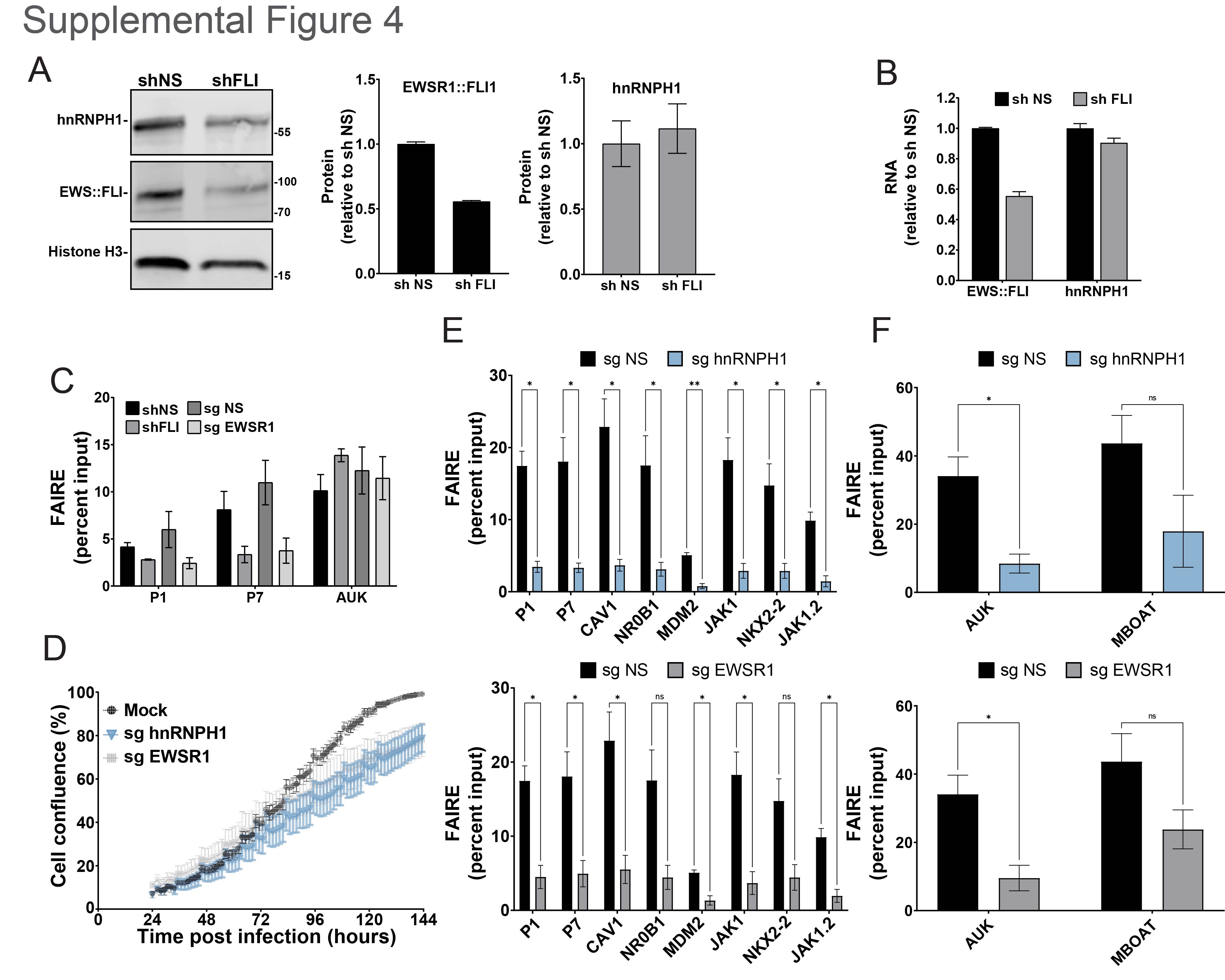

Supplement: Supplementary Figure 4 — (A) Western blot analyses of EWSR1::FLI1 and hnRNPH1 protein following infection with the indicated sh RNAs in A673-CRISPRi cells for 4 days. Left: Representative western blots. Right: Quantification of western blot band intensities for EWSR1::FLI1 and hnRNPH1 normalized to Histone H3 band intensities. Results are shown as the fold change of non-specific guide (sh NS) cells. Error bars represent the standard deviation of three biological replicates. (B) EWSR1::FLI1 and hnRNPH1 expression measured by RT-qPCR in A673-CRISPRi cells following infection with the indicated sh RNAs for 4 days. Results are shown as the fold change of non-specific guide (sh NS) cells. Error bars represent the standard deviation of three biological replicates. (C) FAIRE-qPCR at EWSR1::FLI1-bound and control loci in A673-CRISPRi cells infected with the indicated sg and sh RNAs for 4 days. Results are shown as a fraction of input control. Error bars represent the standard deviation of three biological replicates. (D) Cell proliferation curves by live cell imaging for EWS502-CRISPRi cells infected with the indicated sgRNAs for 4 days. Proliferation was assessed by images captured every 2 hours. Results are shown as the percent confluence. Error bars represent the standard error of the mean of 25 images taken from one well per condition per time point. (E) FAIRE-qPCR at EWSR1::FLI1-bound loci in EWS502-CRISPRi cells infected with the indicated sgRNAs (Top: sg hnRNPH1, Bottom: sg EWSR1) for 4 days. Results are shown as a fraction of input control. Error bars represent the standard error of two biological replicates. (F) FAIRE-qPCR at positive control loci in A673-CRISPRi cells infected with the indicated sgRNAs (Top: sg hnRNPH1, Bottom: sg EWSR1) for 4 days. Results are shown as a fraction of input control. Error bars represent the standard error of three biological replicates. [file Image_4.jpeg]

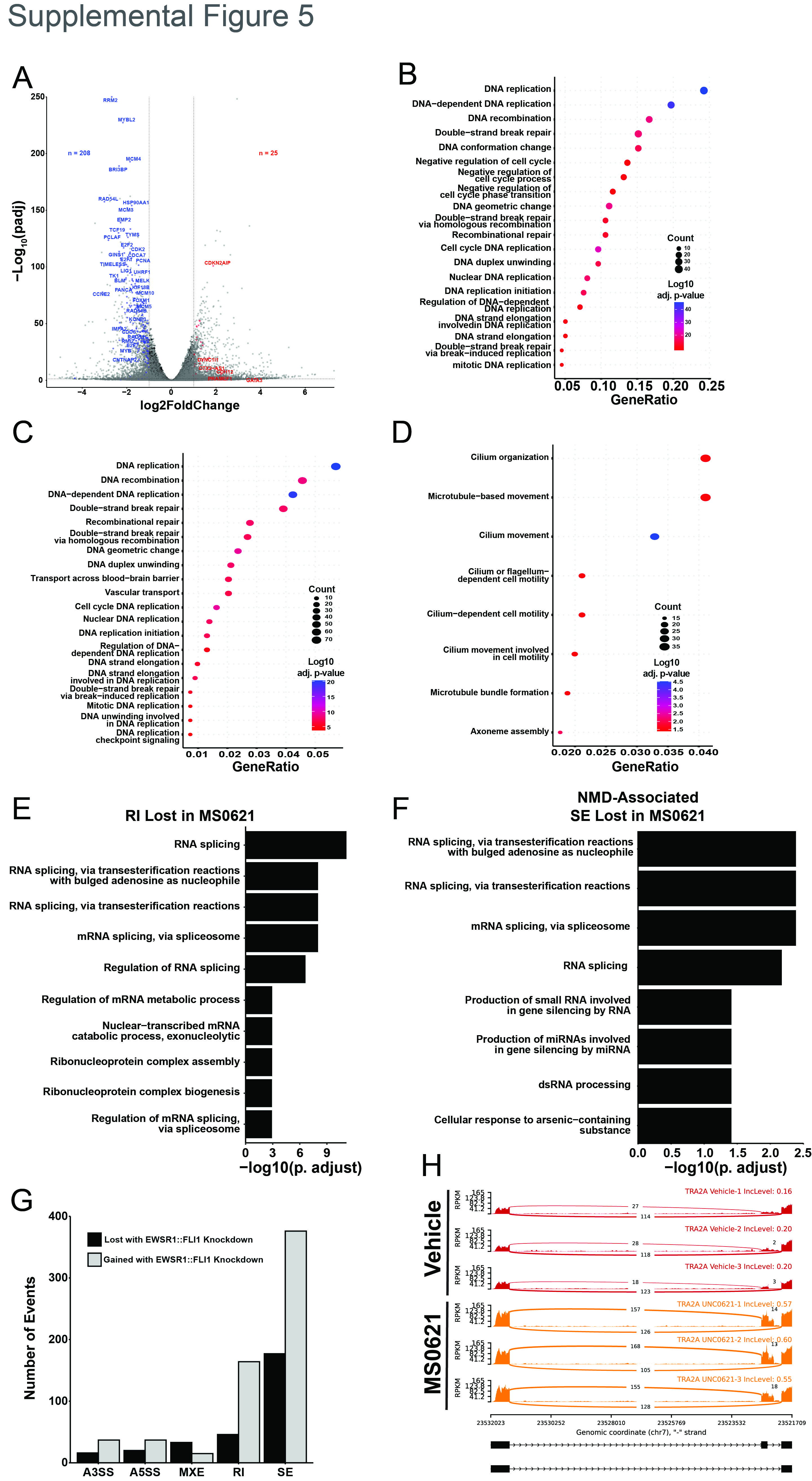

Supplement: Supplementary Figure 5 — (A) Volcano plot of log2 fold change of gene expression in EWS894 cells treated 16 hours with 5 μM MS0621 or vehicle control (DMSO). Colored points represent genes that are both upregulated by EWSR1::FLI1 per Kinsey et al., 2006 and differentially expressed with MS0621 treatment. (B) Enriched Biological Processes GO terms for genes that are both downregulated with MS0621 treatment and upregulated by EWSR1::FLI1. Results shown are the -log10 adjusted p-value for the top 20 enriched GO terms. (C) Enriched Biological Processes GO terms for all genes downregulated with MS0621 treatment. Results shown are the -log10 adjusted p-value for the top 20 enriched GO terms. (D) Enriched Biological Processes GO terms for all genes upregulated with MS0621 treatment. Results shown are the -log10 adjusted p-value for the top 20 enriched GO terms. (E) Enriched Biological Processes GO terms for genes with RI events lost with MS0621 treatment. Results shown are the -log10 adjusted p-value for enriched GO terms. (F) Enriched Biological Processes GO terms for genes with SE events in NMD-associated transcripts events lost with MS0621 treatment. Results shown are the -log10 adjusted p-value for enriched GO terms. (G) Significant differential alternative splicing identified by rMATS in A673 following EWSR1::FLI1 or control knockdown for 48 hr and meeting the additional criteria: supported by at least 20 reads, Inclusion Level Difference > 10%, and FDR < 0.05. Analyzed data were previously published (8). [file Image_5.jpeg]
